# Patient‐ and Areal‐Level Risk Factors Associated With Lung Cancer Mortality in Victoria, Australia: A Bayesian Spatial Survival Analysis

**DOI:** 10.1002/cam4.70293

**Published:** 2024-10-09

**Authors:** Getayeneh Antehunegn Tesema, Rob G. Stirling, Zemenu Tadesse Tessema, Stephane Heritier, Arul Earnest

**Affiliations:** ^1^ School of Public Health and Preventive Medicine Monash University Melbourne Australia; ^2^ Department of Epidemiology and Biostatistics, Institute of Public Health, College of Medicine and Health Sciences University of Gondar Gondar Ethiopia; ^3^ Central Clinical School, Faculty of Medicine, Nursing and Health Sciences Monash University Melbourne Australia; ^4^ Department of Respiratory Medicine Alfred Health Melbourne Australia

**Keywords:** Bayesian analysis, lung cancer mortality, spatial survival models, Victoria, VLCR

## Abstract

**Background:**

In Australia, lung cancer is the leading cause of cancer‐related deaths. In Victoria, the mortality risk is assumed to vary across Local Government Areas (LGAs) due to variations in socioeconomic advantage, remoteness, and healthcare accessibility. Thus, we applied Bayesian spatial survival models to examine the geographic variation in lung cancer survival in Victoria.

**Methods:**

Data on lung cancer cases were extracted from the Victorian Lung Cancer Registry (VLCR). To account for spatial dependence and risk factors of survival in lung cancer patients, we employed a Bayesian spatial survival model. Conditional Autoregressive (CAR) prior was assigned to model the spatial dependence. Deviance Information Criterion (DIC), Watanabe Akaike Information Criterion (WAIC), and Log Pseudo Marginal Likelihood (LPML) were used for model comparison. In the final best‐fitted model, the Adjusted Hazard Ratio (AHR) with the 95% Credible Interval (CrI) was reported. The outcome variable was the survival status of lung cancer patients, defined as whether they survived or died during the follow‐up period (death was our interest).

**Results:**

Our study revealed substantial variations in lung cancer mortality in Victoria. Poor Eastern Cooperative Oncology Group (ECOG) performance status, diagnosed at a regional hospital, Small Cell Lung Cancer (SCLC), advanced age, and advanced clinical stage were associated with a higher risk of mortality, whereas being female, presented at Multidisciplinary Team (MDT) meeting, and diagnosed at a metropolitan private hospital were significantly associated with a lower risk of mortality.

**Conclusion:**

Identifying geographical disparities in lung cancer survival may help shape healthcare policy to implement more targeted and effective lung cancer care services.

## 1 Introduction

According to the 2022 Global Cancer Observatory (GCO) report, over 2.5 million lung cancer cases and 1.8 million deaths were observed [1]. In Australia, lung cancer is the fifth most commonly diagnosed and the top leading cause of cancer mortality, accounting for 9% of all cancer diagnoses [2]. Non‐small cell lung cancer, which is further classified into adenocarcinoma, squamous cell carcinoma, and large cell carcinoma, accounts for about 80%–85% of cases of lung cancer [3]. Small cell lung cancer accounts for 10%–15% of all cases of lung cancer; other lung cancers and lung carcinoid tumors make up the remaining cases. Australia has one of the highest rates of lung cancer survival in the world with a 5‐year survival rate of 22% [4]. In Victoria, it ranks as the fourth most frequently diagnosed cancer and the leading cause of cancer deaths [5]. According to a study conducted in Victoria, Australia, it is anticipated that the incidence of lung cancer in men will increase by 44% to 2515 cases between 2019 and 2028, while the incidence of lung cancer in women will increase by 41% to 1909 cases [6]. The 5‐year survival for lung cancer in Victoria has increased from 10% in 1986–1990 to 27% in 2016–2020 [5]. However, the survival rate is strongly influenced by geographical remoteness and socioeconomic status with the highest mortality in remote and most disadvantaged areas [7, 8, 9].

In Australia (Victoria), studies have demonstrated the geographical and socioeconomic disparity in lung cancer survival [10, 11, 12]. Previous studies have shown that various factors related to the patient, such as age [13, 14], clinical stage of cancer at diagnosis [15], Eastern Cooperative Oncology Group (ECOG) performance status [16], timeliness of care [17], type of cancer [18], and area‐level factors like socioeconomic status [19] and geographic remoteness, have a significant impact on lung cancer mortality.

Despite the huge burden of lung cancer mortality and the crucial role of geographical information in guiding interventions [20, 21], studies on the geographic distribution of the survival status of lung cancer are minimal. Most studies examined the incidence and survival status using conventional survival models [22, 23, 24]. However, this model cannot account for the spatial dependence to obtain efficient parameter estimates nor explore the spatial variation [25]. Thus, patients with lung cancer belonging to the same area share certain unobserved characteristics, which may not be sufficiently accounted for by the observed covariates considered in the conventional regression models.

Prior published studies on cancer performed using the Bayesian spatial survival models [26] demonstrated substantial geographic variation in cancer survival. According to Cramb et al. the cancer survival status in Queensland, Australia has substantial spatial and spatio‐temporal variations [12]. The area‐level risk factors such as area‐level socioeconomic status, remoteness, and patient‐level risk factors contributed to this variation. Data sparsity and confidentiality concerns are the major challenges in estimating lung cancer survival at a small‐area level. Thus, Bayesian spatial survival models' smooth estimates by borrowing strength from neighboring areas and contextual covariates such as census data.

The Bayesian spatial survival models are developed to model geographically indexed survival data [27]. The model accounts for the spatial dependence by assigning Conditional Autoregressive (CAR) priors and a survival model to examine predictors of time to mortality for patients with lung cancer [28]. Unlike other spatial generalized linear models, the Bayesian spatial survival models can allow censored observations in the model.

Thus, the objective of this study was to explore the spatial variation in lung cancer mortality in Victoria using the Bayesian spatial survival model. The extent of the geographic inequalities in the burden of lung cancer mortality has not been explored yet, and therefore, it is important to investigate the difference in state‐based healthcare planning and intervention.

## 2 Methods

### 2.1 Study Design, Setting, and Participants

The data used for this study were obtained from the Victorian Lung Cancer Quality Registry (VLCR) [29]. Victoria is the second largest state in Australia following Western Australia. The spatial unit used was the Local Government Areas (LGAs), defined under the Australian Bureau of Statistics (ABS) Australian Standard Geographic Classification (ASGC). LGA plays a crucial role in managing community needs such as health planning, community services, and infrastructure. Victoria has 79 LGAs, each has its local council responsible for providing services and governing the region.

**FIGURE 1:**
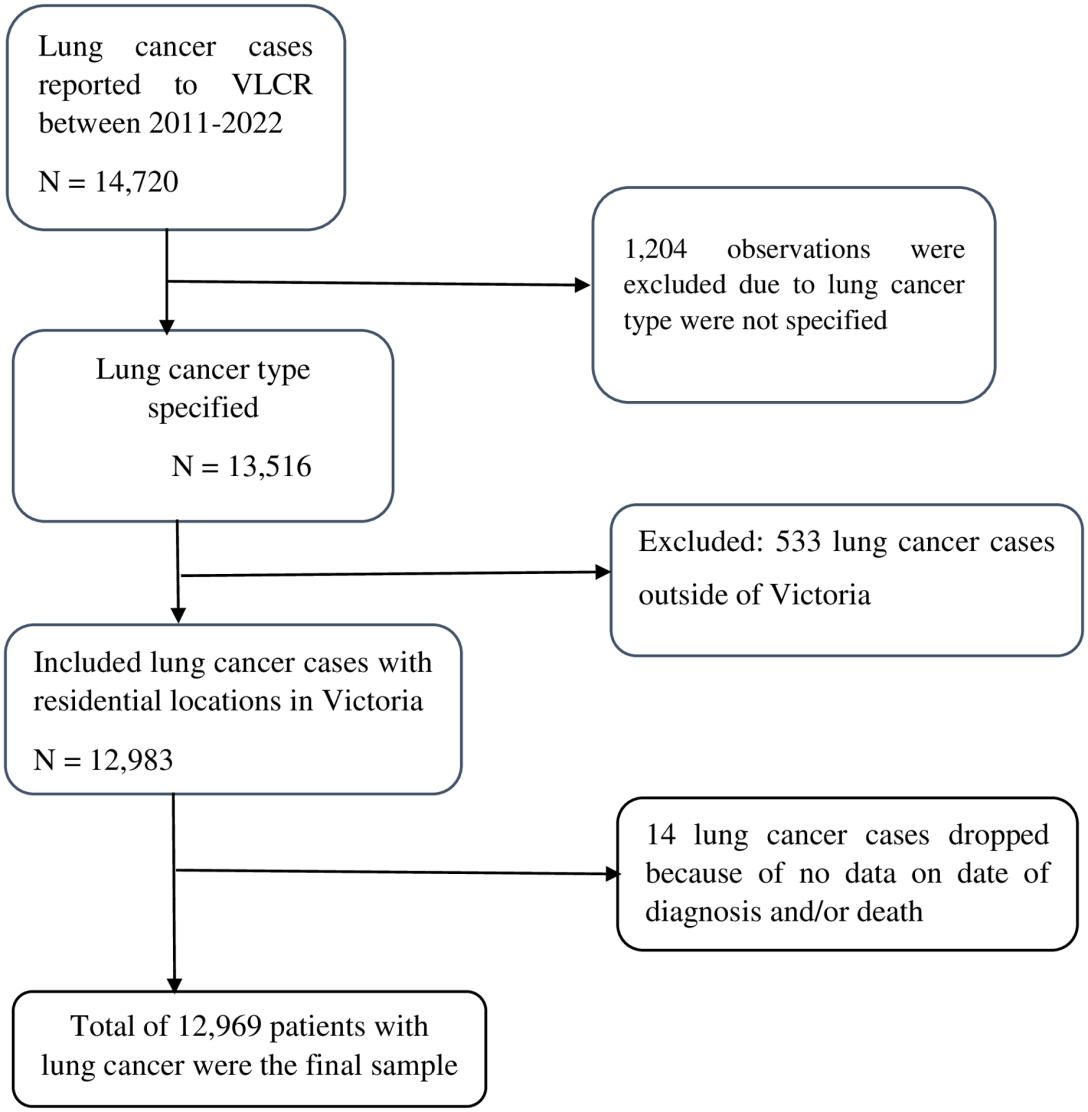
Flow chart to extract study participants for our study.

A retrospective follow‐up study was performed based on lung cancer cases reported to the VLCR between 2011 and 2022. In addition, area‐level socioeconomic status and geographic remoteness were obtained from ABS. VLCR registry is a prospective study that gathers data about patient‐reported outcomes and quality of care [29]. This registry currently captures approximately 90% of new cancer cases in Victoria across 19 healthcare networks and 49 hospitals receiving notifications via an extract of the Victorian Admission Episode Data set from each participating site. Patients aged 18 years or older and newly diagnosed with confirmed primary lung cancer from a participating hospital or healthcare institution were eligible. Secondary lung cancer cases and patients located outside of Victoria were excluded. Finally, a total of 12,969 lung cancer cases that had data on the date of diagnosis and outcome status were considered (Figure 1).

### 2.2 Study Variables

The outcome variable was survival status among patients with lung cancer. Survival time was defined from the date of diagnosis to the date of death or censorship (lost follow or alive till the end of the study). It was calculated in months from the date of diagnosis to the date of death, lost follow‐up, or the end date of the study. The event was recoded as “1” for the patient who died and “0” for a patient who survived. The residential address at diagnosis was geocoded and linked to LGAs.

Independent variables were extracted from the VLCR and ABS. Variables such as age, ECOG performance status, clinical stage at diagnosis, Multidisciplinary Team (MDT) meeting presentation, Systemic Anticancer Therapy (SACT), surgical treatment, referral to diagnosis interval, diagnosis to treatment interval, year of diagnosis, underlying comorbidity, sex, lung cancer type, country of birth, diagnosing hospital, and smoking history were extracted from VLCR. We defined diagnostic and treatment delays as binary. Referral to treatment interval was defined as the time elapsed from the date of referral to diagnosis and the date of diagnosis to treatment, respectively. Referral to a diagnosis interval that exceeded 28 days was considered as a diagnostic delay and referral to a treatment interval that exceeded 42 days was considered as a treatment delay [30].

The ECOG performance status assesses a patient's degree of functioning in terms of their capacity for self‐care, daily activity, and physical capability (walking, working, etc.) [31]. The ECOG performance scale has six scales and we categorized it as good if the ECOG performance scale score was < 2 and poor if the ECOG performance scale score ≥ 2 [32].

Whereas area‐level socioeconomic status measured by the Index of Relative Socioeconomic Advantage and Disadvantage (IRSAD) and geographic remoteness classified based on the Monash Modified Model (MMM) were obtained from ABS [33, 34]. An LGA with a higher IRSAD score indicates a high proportion of relatively advantaged people. We categorized IRSAD into quantiles. Those living within 25% of the most disadvantaged areas were assigned a quartile of 1, whereas those living in 25% of the most advantaged areas were assigned a quartile score of 5. MMM was used to define the geographic remoteness. It has 7 categories such as; MMM 1—metropolitan, MM2—regional, MMM 3—large rural towns, MMM 4—medium rural towns, MMM 5—small rural towns, MMM 6—remote, and MMM 7—very remote areas.

### 2.3 Statistical Analysis

The polygon shapefile data for the Australian map was downloaded from the ABS (https://www.abs.gov.au/statistics/standards/australian‐statistical‐geography‐standard‐asgs‐edition‐3). The Victorian shapefile was then extracted using the statistical software Arc‐GIS 10.8. Our study's spatial unit of analysis was defined using the Victorian boundary shapefile, which included 79 LGAs. The shapefile was loaded directly into R for spatial analysis.

The death and survival of lung cancer patients were defined as events and censors, respectively. The survival time was calculated as the time between diagnosis and death or censoring (measured in months). We used the Kaplan–Meier (KM) failure curve to graph the survival difference across categorical variables and the log‐rank test to compare it objectively. The global‐Schoenfeld residual test revealed a violation of the Proportional Hazard (PH) assumption (*p* < 0.05). When the PH fails, the parametric accelerated failure time model is used as an alternative approach.

Let $T_{\mathrm{ij}}$ denote the survival time (time to death or censor) for lung cancer patient *j* in LGA *i*, $x_{\mathrm{ij}}$ is the vector of covariates to $T_{\mathrm{ij}}$, where *j* = 1, 2, …, 12,969, and *i* = 1, 2, 3, …, 79. The parametric accelerated failure time model is formulated as follows;

$$\log\left(T_{\mathrm{ij}}\right)=\mu +{\mathrm{\beta x}}_{\mathrm{ij}}+{\rho \varepsilon }_{\mathrm{ij}}$$

where $\beta =\left(\beta_{0},\beta_{1},\beta_{2},\ldots ,\beta_{n}\;\right)$ is the vector of regression coefficients, $\varepsilon_{\mathrm{ij}}$ is the residuals, and μ and ρ are the shape and scale parameters, respectively.

Based on previous research demonstrating spatial variation in lung cancer survival across areas, we hypothesized that lung cancer survival would be spatially correlated, and thus ignoring this could affect regression parameter estimates. In addition, we confirmed a significant difference in median survival time between LGAs. As a result, we extended the survival model to the Bayesian spatial survival model, which captures spatial dependence by assigning prior CAR distributions. The Bayesian spatial survival model was fitted using R‐version 4.3.3. statistical software based on the spBayesSurv package [27]. We fitted the Bayesian spatial survival model to explore lung cancer survival across LGAs in Victoria.

We applied the spatial random effect in the parametric survival model to estimate the survival difference in lung cancer across LGAs and improve risk estimates. As a result, we extend the traditional parametric survival model to the spatial parametric survival model. We used the Besag York Mollie (BYM) with a CAR model to account for spatial dependence [35]. The parametric survival spatial model was formulated as [36];

$$\log\left(T_{\mathrm{ij}}\right)=\mu +{\mathrm{\beta x}}_{\mathrm{ij}}+{\rho \varepsilon }_{\mathrm{ij}}+W_{i}$$

where $W_{i}$ is the spatial random effect (LGA‐specific random effect, *i* = 1, 2, 3, …, 79). It was defined by an adjacency matrix based on the first‐order Queens contiguity generated using the spdep R‐package. This adjacency matrix was used as a parameter of the CAR prior distribution in the model formulation. Queen contiguity was used to define the spatial weight matrix.

For the structured spatial random effect, the CAR model developed by Besag was used:

$$\left[u_{i}|u_{j},i\neq j,{\tau_{u}}^{2}\right]\sim N\;\left({ū}_{i,}{\tau_{i}}^{2}\right)$$

where [37].

$${\bar{u}}_{i}\;=\;\frac{1}{\sum_{j}w_{\mathrm{ij}}}\sum u_{j}w_{\mathrm{ij}}$$

$$\tau_{i}^{2}=\frac{\tau_{u}^{2}}{\sum_{j}w_{\mathrm{ij}}}$$

*w*_*ij*_ = 1 if *i* and *j* are adjacent, or 0 otherwise. We assign 1 for adjacent LGAs in the adjacency matrix and 0 if they were not neighbors.

We considered three baseline distributions such as Weibull, log‐normal, and log‐logistic distributions. Altogether, we have fitted seven different models combining the above‐mentioned baseline distributions, spatial dependence, and covariates within the Bayesian framework.

### 2.4 Bayesian Inference

The model parameters were estimated by approximating the posterior distribution of models based on the Markov Chain Monte Carlo (MCMC) simulation algorithm in a Bayesian framework. All the models had a burn‐in of 5000 iterations, then monitored for 10,000 iterations, keeping every fourth iteration to reduce the autocorrelation in the posterior estimates. The model fit was assessed using the Cox–Snell residual plot, and model convergence was assessed using a trace plot. Three models were compared based on the Deviance Information Criterion (DIC), Watanabe Akaike Information Criterion (WAIC), and Log Pseudo Marginal Likelihood (LPML) [27]. The smallest DIC and WAIC, and largest LPML values indicate model improvement. In the final best‐fitted model, a posterior estimate of the spatial variance and the covariates with their corresponding 95% Credible Intervals (CrI) were reported to declare the statistical significance and strength of the association.

## 3 Results

### 3.1 Patient Characteristics

Among the 12,969 patients diagnosed with lung cancer, the majority were males (56.29%), Australian‐born (61.15%), Small Cell Lung Cancer (SCLC) (88.04%), and clinical stage IV (45.14%). Regarding the ECOG performance status, about 6742 (51.99%) had a good performance at diagnosis. Most of the patients (85.49%) were ever smokers and nearly two‐thirds (65.75%) of the cases were discussed at the MDT meetings. More than two‐thirds (70.61%) of the patients belonged to metropolitan areas followed by small rural areas (14.74%). The mean age of patients at diagnosis was 69.32 ± 10.67 years and more than one‐third of the patients (35.60%) were in the 70–79 years age group (Table 1).

**TABLE 1:** Baseline characteristics of lung cancer patients in Victoria, Australia (*n* = 12,969).

Characteristics	Frequency (*n*)	Percentage
Sex		
Male	7300	56.29
Female	5669	43.71
Age (in years)		
< 60	2287	17.63
60–69	3871	29.85
70–79	4617	
≥ 80	2194	16.92
Type of cancer		
SCLC	11,418	88.04
NSCLC	1551	11.96
Clinical stage of cancer		
Stage I	1759	13.56
Stage II	1010	7.79
Stage III	2037	15.71
Stage IV	5854	45.14
Not stated	2309	17.80
ECOG performance status		
Good	6742	51.99
Poor	1892	14.59
Not stated	4335	33.43
Smoking history		
Ever	11,087	85.49
Never	1517	11.70
Not stated	365	2.81
Presented at MDT meeting		
No	4442	34.25
Yes	8527	65.75
Time of diagnosis		
Timely	7997	61.66
Delayed	3591	27.70
Not stated	1380	10.64
Time of treatment		
Timely	4797	36.99
Delayed	5039	38.85
Not stated	3133	24.16
Receiving surgery		
No	9511	73.34
Yes	3116	24.03
Not stated	342	2.64
Country of birth		
Australian	7931	61.15
Others	5038	38.85
Diagnosing hospital		
Metropolitan public	8148	62.83
Metropolitan private	2166	16.70
Regional	2655	20.47
*Comorbidity status*		
Diabetes mellitus		
No	10,587	81.63
Yes	2382	18.37
Myocardial infarction		
No	10,995	84.78
Yes	1974	15.22
Respiratory comorbidity		
No	9293	71.66
Yes	3676	28.34
Renal insufficiency		
No	12,332	95.09
Yes	637	4.91
Neoplastic comorbidity		
No	10,374	79.99
Yes	2595	20.01
MMM remoteness classification		
Metropolitan	9158	70.61
Regional	866	6.68
Large rural	454	3.50
Medium rural	542	4.18
Small rural	1912	14.74
Remote	37	0.29
IRSAD		
1st quartile (least advantaged)	2904	22.39
2nd quartile	1877	14.47
3rd quartile	2180	16.81
4th quartile	2473	19.07
5th (most advantaged)	3535	27.26

Abbreviations: ECOG, Eastern Cooperative Oncology Group; IRSAD, Index of Relative Socioeconomic Advantage and Disadvantage; MMM, Monash Modified Model; NSCLC, Non‐small Cell Lung Cancer; SCLC, Small Cell Lung Cancer.

Of the 12,969 lung cancer patients, 8278 (63.83%) died and 4691 (36.17%) were alive. More than two‐thirds (67.79%) and 3329 (58.72%) of males and females died, respectively. Age, sex, lung cancer type, clinical stage, ECOG performance status, smoking history, MDT meeting presentation, time of diagnosis and treatment, diagnosing hospital, receiving surgery, and country of birth had a statistically significant association with lung cancer mortality (Table 2). In addition, the KM cumulative failure curve revealed that there was a significant survival difference across age, sex, lung cancer type, smoking history, ECOG performance status, MDT meeting presentation, diagnosing hospital, clinical stage, time of diagnosis, and treatment (Figure S1).

**TABLE 2:** Distribution of lung cancer mortality by baseline characteristics.

Variables	Status		*p*
	Alive (%)	Died (%)	
Sex			
Male	2351 (32.21)	4949 (67.79)	< 0.001
Female	2340 (41.28)	3329 (58.72)	
Age			
< 60	950 (41.54)	1337 (58.46)	< 0.001
60–69	1498 (38.70)	2373 (61.30)	
70–79	1727 (37.41)	2890 (62.59)	
≥ 80	516 (23.52)	1678 (76.48)	
Lung cancer type			
NSCLC	4389 (38.44)	7029 (61.56)	< 0.001
SCLC	302 (19.47)	1249 (80.53)	
Clinical stage			
Stage I	1310 (74.47)	449 (25.53)	< 0.001
Stage II	555 (54.95)	455 (45.05)	
Stage III	782 (38.39)	1255 (61.61)	
Stage IV	1072 (18.31)	4782 (81.69)	
Not stated	972 (42.10)	1337 (57.90)	
ECOG performance status			
Good	2818 (41.80)	3924 (58.20)	< 0.001
Poor	322 (17.02)	1570 (82.98)	
Not stated	1551 (35.78)	2784 (64.22)	
Smoking history			
Ever	3821 (34.46)	7266 (65.54)	< 0.001
Never	775 (51.09)	742 (48.91)	
Not stated	95 (26.03)	270 (73.97)	
Presented at MDT meeting			
No	1123 (25.28)	3319 (74.72)	< 0.001
Yes	3568 (41.84)	4959 (58.16)	
Time to diagnosis			
Timely	2406 (30.09)	5591 (69.91)	< 0.001
Delayed	1883 (52.42)	1709 (47.58)	
Not stated	402 (29.13)	978 (70.87)	
Time to treatment			
Timely	1631 (34.00)	3166 (66.00)	< 0.001
Delayed	2507 (49.75)	2532 (50.25)	
Not stated	553 (17.65)	2580 (82.35)	
Receiving surgery			
No	2294 (24.12)	7217 (75.88)	< 0.001
Yes	2306 (74.01)	810 (25.99)	
Not stated	91 (26.61)	251 (73.39)	
Place of birth			
Australian	2813 (35.47)	5118 (64.53)	0.037
Other/not stated	1878 (37.28)	3160 (62.72)	
Diagnosing hospital			
Metropolitan public	2919 (35.82)	5229 (64.18)	< 0.001
Metropolitan private	941 (43.44)	1225 (56.56)	
Regional	831 (31.30)	1824 (68.70)	
MMM remoteness classification			
Metropolitan	3309 (36.13)	5849 (63.87)	0.880
Regional	326 (37.64)	540 (62.36)	
Large rural	170 (37.44)	284 (62.56)	
Medium rural	188 (34.69)	354 (65.31)	
Small rural	685 (35.83)	1227 (64.17)	
Remote	13 (35.14)	24 (64.86)	
IRSAD			
1st quartile (least advantaged)	1046 (36.02)	1858 (63.98)	0.269
2nd quartile	681 (36.28)	1196 (63.72)	
3rd quartile	793 (36.38)	1387 (63.62)	
4th quartile	935 (37.81)	1538 (62.19)	
5th (most advantaged)	1236 (34.96)	2299 (65.04)	
Overall	4691 (36.17)	8278 (63.83)	

Abbreviations: ECOG, Eastern Cooperative Oncology Group; IRSAD, Index of Relative Socioeconomic Advantage and Disadvantage; MMM, Monash Modified Model; NSCLC, Non‐small Cell Lung Cancer; SCLC, Small Cell Lung Cancer.

### 3.2 Posterior Summaries of Covariates and Spatial Frailty

The best‐fitted model was the model containing the spatial frailty term and covariates like age, sex, smoking status, time of treatment, ECOG performance status, type of lung cancer, MDT meeting presentation, and diagnosing hospital (Table S1). Based on the bivariable Bayesian spatial survival analysis, we incrementally built the model by incorporating statistically and clinically relevant covariates. Using DIC, WAIC, and LPML as our criteria, we retained the covariate that had the lowest DIC value while gradually adding the next most significant covariate. To avoid model overfitting, we performed sensitivity analysis (i.e., including area‐level covariates like IRSAD and geographic remoteness). The model constructed without these covariates had a lower DIC and WAIC and a higher LPML.

The trace plots of the Markov Chain were used to assess the proper mixing of the parameters. They were stationary, indicating convergence (Figure S2). The Cox–Snell residuals, which are equivalent to normal probability plots, test the model's reliability. The Cox–Snell plot revealed nearly straight hazard plots, with slope one, indicating a good model (Figure S3). In the final best‐fitted model, sex, age, clinical stage at diagnosis, ECOG performance status, MDT meeting presentation, lung cancer type, diagnosing hospital, and smoking history were found significant predictors of lung cancer mortality.

The hazard of mortality among female lung cancer patients was lowered by 21% (Adjusted Hazard Ratio [AHR] = 0.79, 95% CrI: 0.74, 0.85) than male patients. Compared to clinical stage I, about 2.20 (AHR = 2.20, 95% CrI: 1.89, 2.56), 4.61 (AHR = 4.61, 95% CrI: 4.03, 5.28), and 12.43 (AHR = 12.43, 95% CrI: 10.99, 13.99) times higher mortality in stage II, stage III and stage IV were observed, respectively. The hazard of mortality among patients who had poor ECOG performance at diagnosis was 2.45 times (AHR = 2.45, 95% CrI: 2.22, 2.68) higher compared to patients with good ECOG performance at diagnosis. The hazards of mortality among patients with lung cancer whose cases were discussed at the MDT meeting were lowered by 31% (AHR = 0.69, 95% CrI: [0.64, 0.75]) than those who were not discussed at the MDT meeting.

The hazard of mortality among patients diagnosed at metropolitan private hospitals was lowered by 41% (AHR = 0.57, 95% CrI: 0.51, 0.63) compared to patients who were diagnosed at metropolitan public hospitals. Whereas, lung cancer patients diagnosed at regional hospitals had 1.19 times (AHR = 1.19, 95% CrI: 1.06, 1.33) higher hazard of mortality compared to those diagnosed at metropolitan public hospitals. SCLC cases had 1.21 times (AHR = 1.21, 95% CrI: 1.10, 1.33) higher hazard of mortality compared to NSCLC cases. Compared to ever smokers, the hazards of mortality among never smokers were lowered by 51% (AHR = 0.49, 95% CrI: 0.44, 0.55) (Table 3).

**TABLE 3:** Bivariable and multivariable Bayesian spatial log‐logistic survival analysis of predictors of lung cancer mortality.

Predictors	Bivariable Bayesian spatial survival model (HR with 95% CrI)	Multivariable Bayesian spatial survival model (HR with 95% CrI)
Sex		
Male	1	1
Female	0.71 (0.66, 0.76)	0.79 (0.74, 0.85)*
Age (in years)		
< 60	1	1
60–69	1.15 (1.05, 1.27)	1.17 (1.06, 1.29)*
70–79	1.29 (1.18, 1.41)	1.43 (1.31, 1.57)*
≥ 80	2.34 (2.10, 2.60)	1.96 (1.74, 2.21)*
Clinical stage at diagnosis		
Stage I	1	1
Stage II	2.30 (1.97, 2.70)	2.20 (1.89, 2.56)*
Stage III	4.87 (4.27, 5.55)	4.61 (4.03, 5.28)*
Stage IV	15.97 (14.14, 18.05)	12.43 (10.99 13.99)*
Not stated	4.99 (4.36, 5.72)	3.65 (3.19, 4.18)*
ECOG performance status		
Good	1	1
Poor	3.88 (3.54, 4.26)	2.45 (2.22, 2.68)*
Not stated	1.48 (1.38, 1.58)	1.28 (1.19, 1.38)*
Time of treatment		
Timely	1	1
Delayed	0.95 (0.89, 1.02)	1.07 (0.99, 1.15)
Not stated	9.01 (8.12, 9.99)	6.79 (5.79, 7.89)
Presented at MDT meeting		
No	1	1
Yes	0.46 (0.43, 0.49)	0.69 (0.64, 0.75)*
Diagnosing hospital		
Metropolitan public	1	1
Metropolitan private	0.65 (0.59, 0.71)	0.57 (0.51, 0.63)*
Regional	1.42 (1.28, 1.60)	1.19 (1.06, 1.33)*
Lung cancer type		
NSCLC	1	1
SCLC	1.92 (1.76, 2.10)	1.21 (1.10, 1.33)*
Smoking history		
Ever smoker	1	1
Never smoker	0.47 (0.42, 0.52)	0.49 (0.44, 0.55)*
Not stated	1.45 (1.20, 1.75)	0.88 (0.73, 1.07)
MMM remoteness classification		
Metropolitan	1	
Regional	1.05 (0.90, 1.22)	
Large rural	0.97 (0.78, 1.19)	
Medium rural	0.96 (0.79, 1.16)	
Small rural	0.99 (0.86, 1.14)	
Remote	1.27 (0.70, 2.37)	
IRSAD		
1st quartile (least advantaged)	1	
2nd quartile	0.95 (0.85, 1.05)	
3rd quartile	0.98 (0.88, 1.09)	
4th quartile	0.90 (0.81, 1.01)	
5th (most advantaged)	0.93 (0.83, 1.05)	

Abbreviations: CrI, Credible Interval; ECOG, Eastern Cooperative Oncology Group; HR, Hazard Ratio; IRSAD, Index of Socioeconomic Advantage and Disadvantage; MDT, Multidisciplinary Team; MMM, Monash Modified Model; NSCLC, Non‐small Cell Lung Cancer; SCLC, Small Cell Lung Cancer.

* indicates 95% CrI does not include 1.

**FIGURE 2:**
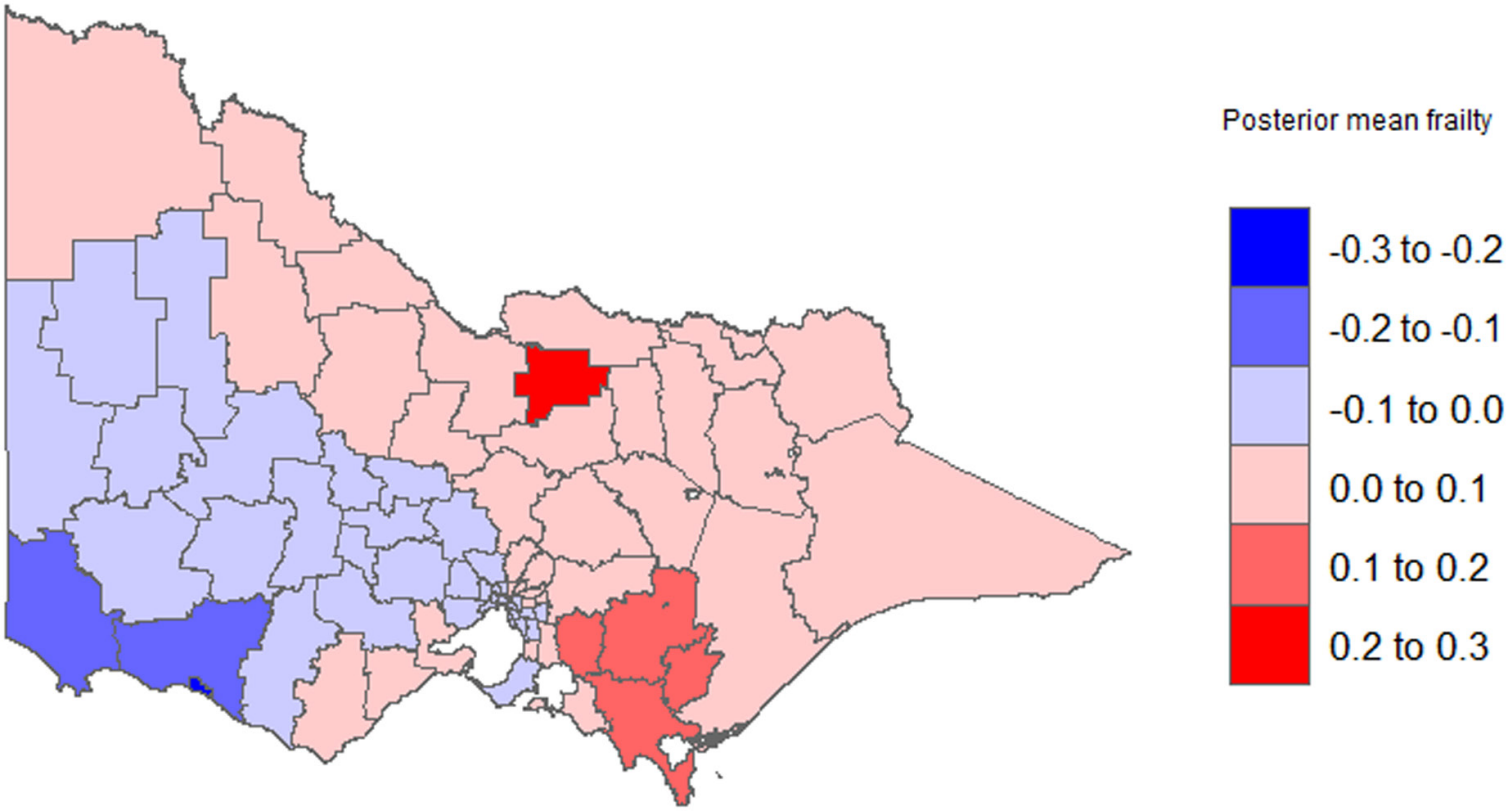
Map of unadjusted smoothed posterior mean frailties; larger frailties mean high mortality rate.

**FIGURE 3:**
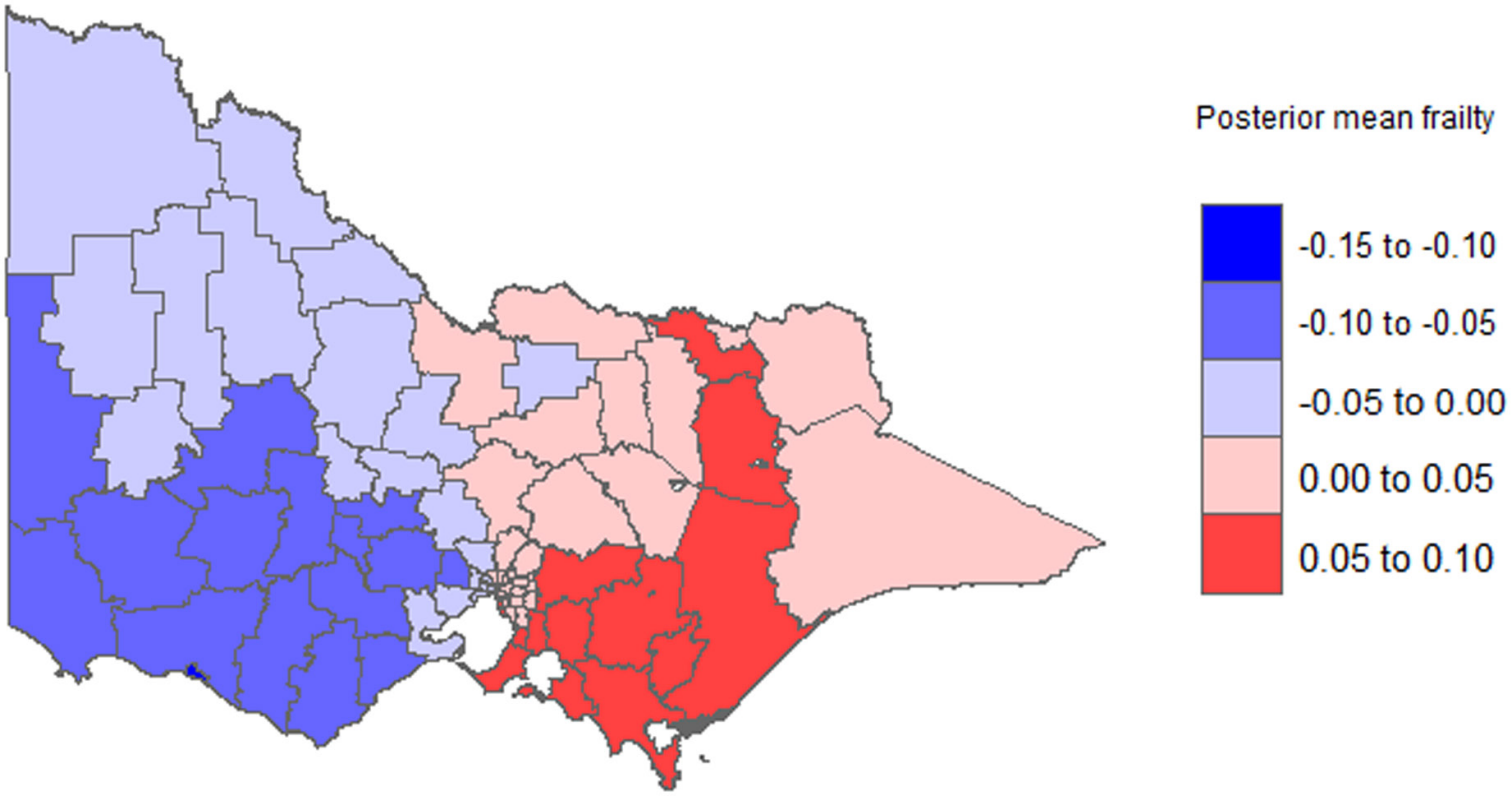
Map of fully adjusted smoothed posterior mean frailties; larger frailties mean high mortality rate.

The variance of posterior frailty of the spatial term was 0.015 (95% CrI: 0.0182, 0.45), which was statistically significant with a 95% credible interval (Tables S2 and S4). We mapped the posterior means of spatial frailties in which each LGA has specific frailties. Figure 2 and Figure 3 show the unadjusted and fully adjusted spatially smoothed posterior mean frailties. The maps showed higher mean frailties or higher hazards of mortality (LGAs with highly dense colors) located in southeastern parts of Victoria. Lung cancer patients from these LGAs (Cardinia, south Gippsland, Latrobe (vic.), Baw Baw, and Greater Shepperton) were shown to have the highest hazard of mortality adjusted for the effects of covariates. Whereas, LGAs in southwestern parts of Victoria (Warrnambool, Moyne, and Glenelg) had the lowest posterior mean frailties (LGAs with less dense colors). Lung cancer patients from these LGAs had the lowest risk of mortality.

## 4 Discussion

Our study investigated the spatial disparities in lung cancer survival that exist in Victoria's LGAs. The spatial frailty log‐logistic model was used to identify the significant predictors of lung cancer mortality. In our study, including spatial frailty terms significantly improves model fit, demonstrating evidence for the spatial variation of lung cancer survival within the state. Our study, therefore, analyzed spatial variation of the time to death of patients with lung cancer using the Bayesian spatial log‐logistic model.

Our study found significant variation in lung cancer survival in Victoria. The distribution coincides with the distribution of smoking prevalence throughout Victoria. LGAs in Northeastern Victoria have a higher risk for mortality probably due to high smoking rates. To reduce geographic disparities in lung cancer mortality in Victoria and improve lung cancer survival in high‐risk areas, policy action is required, ranging from early detection to increased access to quality cancer care. Equitable access to quality cancer care services in Victoria should be ensured by decentralizing cancer care wherever feasible. This finding supports the previously reported finding by Australia, this could be due to the demographical composition and socioeconomic variations across areas. High‐risk LGAs may be due to environmental exposures such as asbestos exposure, which could increase their risk of different comorbidities and, in turn, increase their risk of mortality [38].

After adjusting for the spatial structure, sex, age, clinical stage at diagnosis, ECOG performance status, smoking history, lung cancer type, type of diagnosing hospital, and MDT meeting presentation were found to be significant predictors of lung cancer mortality. Female lung cancer patients had lower hazards of mortality than males. This finding was consistent with studies reported in Turkey [39] and Saudi [24]. It could be attributed to sex‐specific hormonal, behavioral, and genetic variations. Evidence demonstrated that hormonal influences, such as estrogen, play a protective role in lung cancer progression by influencing cancer growth and treatment response [40]. Advanced‐age patients were at a higher risk of death than those under the age of 60. It was supported by previous research findings [41, 42]. Older patients with lung cancer are more vulnerable to acquiring comorbidities [14, 43], which can complicate cancer treatment and increase the risk of mortality. Also, the risk of cancer treatment intolerance rises with age, potentially limiting cancer treatment options and efficacy [44].

Another important predictor of mortality was the clinical stage of diagnosis. Patients diagnosed at an advanced stage had a higher risk of death than those diagnosed at an earlier stage. It was consistent with studies reported in California [45], and Denmark [46]. When cancer advances, it may spread to other organs, complicating cancer treatment [47]. Although lung cancer can be curable if diagnosed at an early stage, advanced cases respond poorly to cancer treatment [48], potentially increasing the risk of mortality [49]. The baseline ECOG performance status was significantly associated with mortality in lung cancer patients. Patients with good ECOG performance status at diagnosis had a lower risk of death than those with poor ECOG performance status. It was consistent with previous research from Israel [16] and Japan [50]. It is known that good ECOG performance status reflects an improved quality of life and is free of underlying comorbidities so they tolerate aggressive anticancer treatment and are more likely to have a quick recovery and positive treatment outcomes [51]. Likewise, good performance status is associated with early‐stage lung cancer, which is more likely to be curable and has a longer survival time.

Our study found that non‐smokers had a lower risk of mortality than ever smokers. It was consistent with studies reported in the United States [13, 52]. It may be that the carcinogens in cigarettes exacerbate the progression of lung cancer by damaging pulmonary cells and causing diseases other than lung cancer such as heart disease, chronic inflammatory lung disease, and other cancers [53]. Evidence suggests that smokers are more likely to have poor cancer treatment outcomes and complications [54]. Consistent with previous research findings in Sweden [55], MDT meeting discussion of lung cancer cases reduces the risk of mortality among lung cancer [56]. It could be attributed to MDT meetings for cancer care, which facilitate timely and evidence‐based cancer management [57]. There is evidence that MDT presentation has a positive impact on cancer treatment outcomes such as survival and timely treatment receipt [56, 58]. In Australia, MDT meeting presentation of lung cancer cases is included as a component of lung cancer care to improve diagnostic and treatment quality [59]. Consistent with previous studies [60, 61, 62], SCLC had a higher risk of mortality than NSCLC. It could be due to the fact that SCLC is very aggressive and metastasizes to other sites more quickly than NSCLC, giving SCLC a reduced likelihood of early diagnosis and treatment [63]. Lung cancer patients diagnosed in regional hospitals had a higher mortality rate than those in metropolitan hospitals. It could happen that patients diagnosed in regional hospitals do not have timely or equitable access to specialized cancer treatment services or advanced diagnostic modalities, leading to a higher risk of mortality.

The strength of our study includes the use robust statistical model (Bayesian spatial parametric survival model) that has the benefit of analyzing the georeferenced survival data to obtain reliable parameter estimates by accounting for censored information and borrowing strength from adjacent areas. In addition, it was based on a large real‐world evidence registry dataset and this enhanced the external validity of the findings. Our study also has some limitations such as the unavailability of data on the change in location after diagnosis. If patients moved to another LGA after diagnosis, it could influence the reliability of parameter estimates and posterior frailty. Despite the genomic era has transformed lung cancer treatment from a universal strategy to a more personalized, precision‐based paradigm, increasing survival rates and quality of life for many patients. Such data were not available in VLCR. This may be possible in a future study when the data collection has matured. All data registries are restricted by resource limitations creating the need for the most parsimonious approach to data collection. Many molecular registries are significantly limited by the absence of key clinical patient data including weight loss, performance status, and comorbidities. The future of lung cancer registry reporting will entail further opportunities for data linkage between clinical quality registries, molecular data repositories, tissue banks, and administrative datasets to optimize lung cancer outcome reporting. Besides, selection bias may exist because data from 90% of newly diagnosed lung cancer cases in Victoria are included in the VLCR. Moreover, the MCMC estimation method was computationally intensive and took several hours to run due to the complexity of the model. Furthermore, the majority of the patients were from metropolitan hospitals. The underrepresentation of patients from regional and remote hospitals can indeed skew the results and may not provide a complete picture of the healthcare needs in those areas.

## 5 Conclusions

In conclusion, substantial geographic variations in lung cancer survival were observed in Victoria. Clinical stage, sex, age, ECOG status, type of diagnosing hospital, type of cancer, and smoking were found significant predictors of mortality among lung cancer. The model accounting for the spatial dependence and covariates performed better. The risk of mortality was increased with advanced age, advanced stage, SCLC, regional hospitals, poor ECOG status, and smoking. Identifying the geographical inequalities in lung cancer survival would assist in implementing targeted and improved lung cancer care services.

## Author Contributions

**Getayeneh Antehunegn Tesema:** conceptualization (equal), data curation (equal), formal analysis (equal), investigation (equal), methodology (equal), software (equal), validation (equal), visualization (equal), writing – original draft (equal), writing – review and editing (equal). **Rob G. Stirling:** conceptualization (equal), supervision (equal), validation (equal), visualization (equal), writing – review and editing (equal). **Zemenu Tadesse Tessema:** conceptualization (equal), data curation (equal), formal analysis (equal), methodology (equal), validation (equal), visualization (equal), writing – review and editing (equal). **Stephane Heritier:** conceptualization (equal), data curation (equal), formal analysis (equal), investigation (equal), methodology (equal), validation (equal), visualization (equal), writing – review and editing (equal). **Arul Earnest:** conceptualization (equal), data curation (equal), formal analysis (equal), methodology (equal), software (equal), supervision (equal), validation (equal), visualization (equal), writing – original draft (equal), writing – review and editing (equal).

## Ethics Statement

The data collection and analysis were conducted in accordance with the principle of the Declaration of Helsinki. The retrospective study protocol was reviewed and approved by the Human Research Ethics Committee of Monash University (approval number: 35288). Hence, written informed consent was waived because of the retrospective nature of the study.

## Conflicts of Interest

The authors declare no conflicts of interest.

## Supporting information


Figure S1.

Figure S2.

Figure S3.

Data S1.

## Data Availability

The data that support the findings of this study are available on request from the corresponding author. The data are not publicly available due to privacy and ethical restrictions.
