# Supplementary material for: Patient‐ and Areal‐Level Risk Factors Associated With Lung Cancer Mortality in Victoria, Australia: A Bayesian Spatial Survival Analysis
Source: Cancer Med. 2024 Oct 9;13(19):e70293. doi: 10.1002/cam4.70293 (PMC11462597; doi:10.1002/cam4.70293)
Supplement: Supplementary file 1 — Figure S1. [file CAM4-13-e70293-s004.pdf]

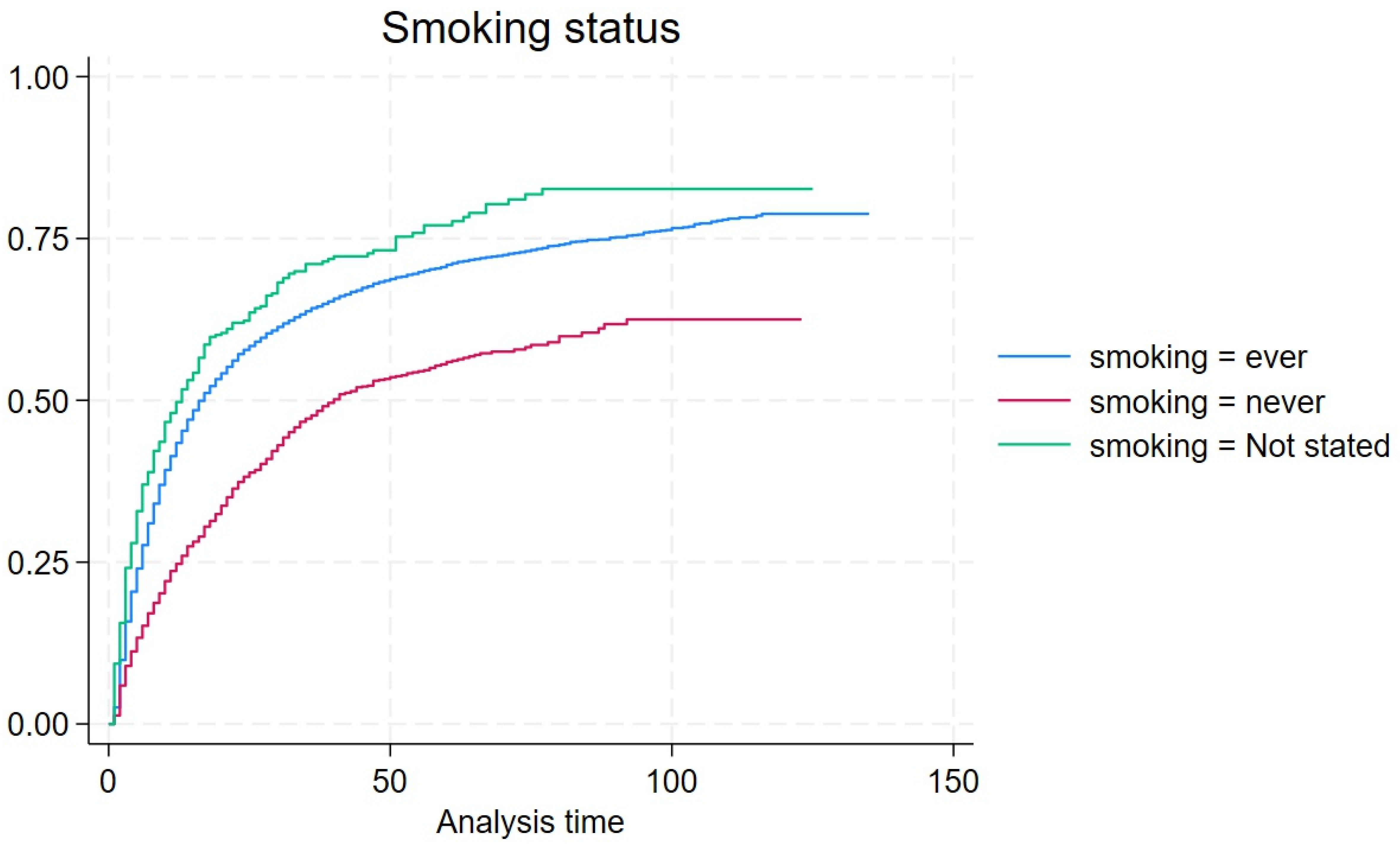

Smoking status
1.00
0.75
0.50
0.25
0.00
0
50
100
150
Analysis time
smoking = ever
smoking = never
smoking = Not stated

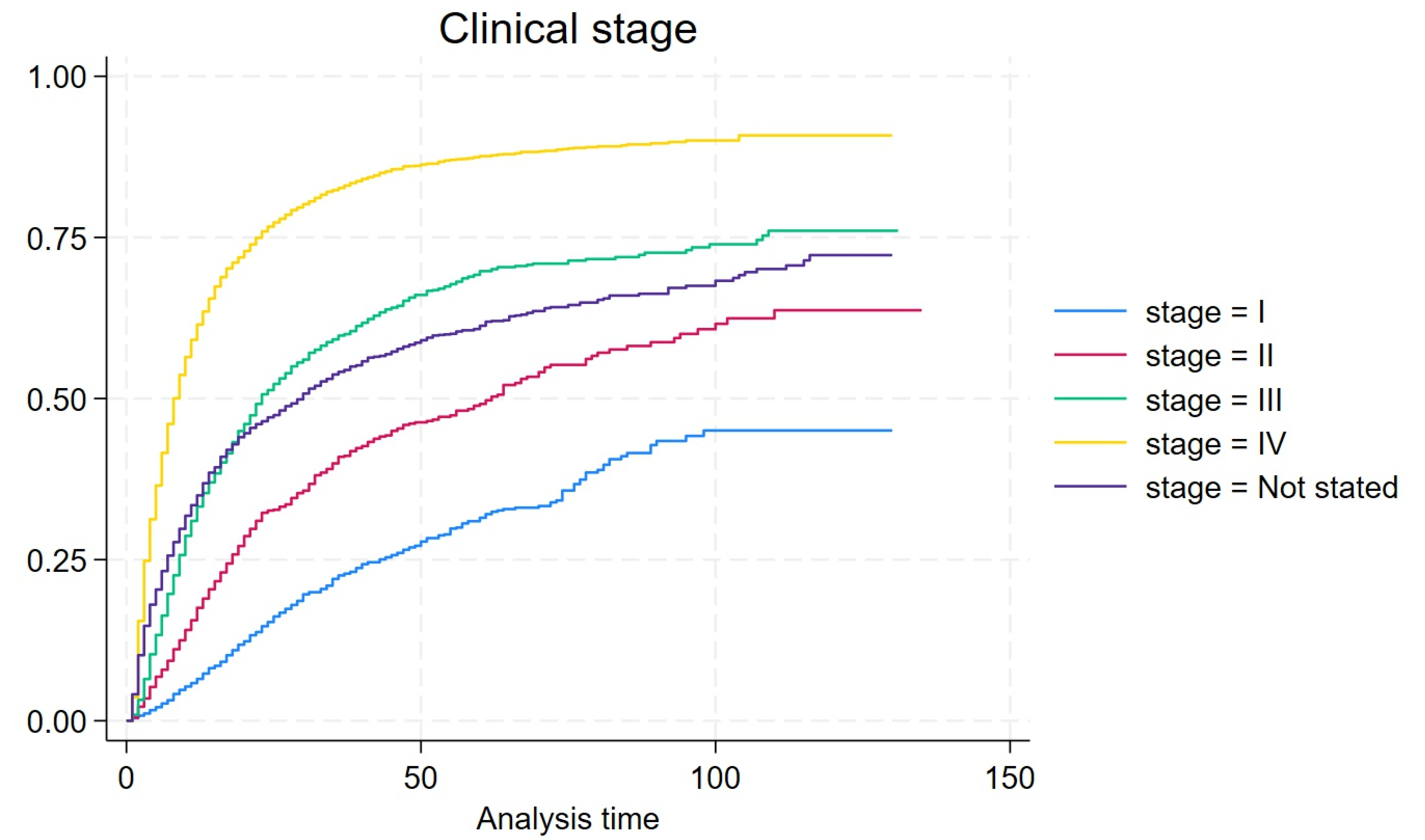
Clinical stage
1.00
0.75
0.50
0.25
0.00
0
50
100
150
Analysis time
stage = I
stage = II
stage = III
stage = IV
stage = Not stated

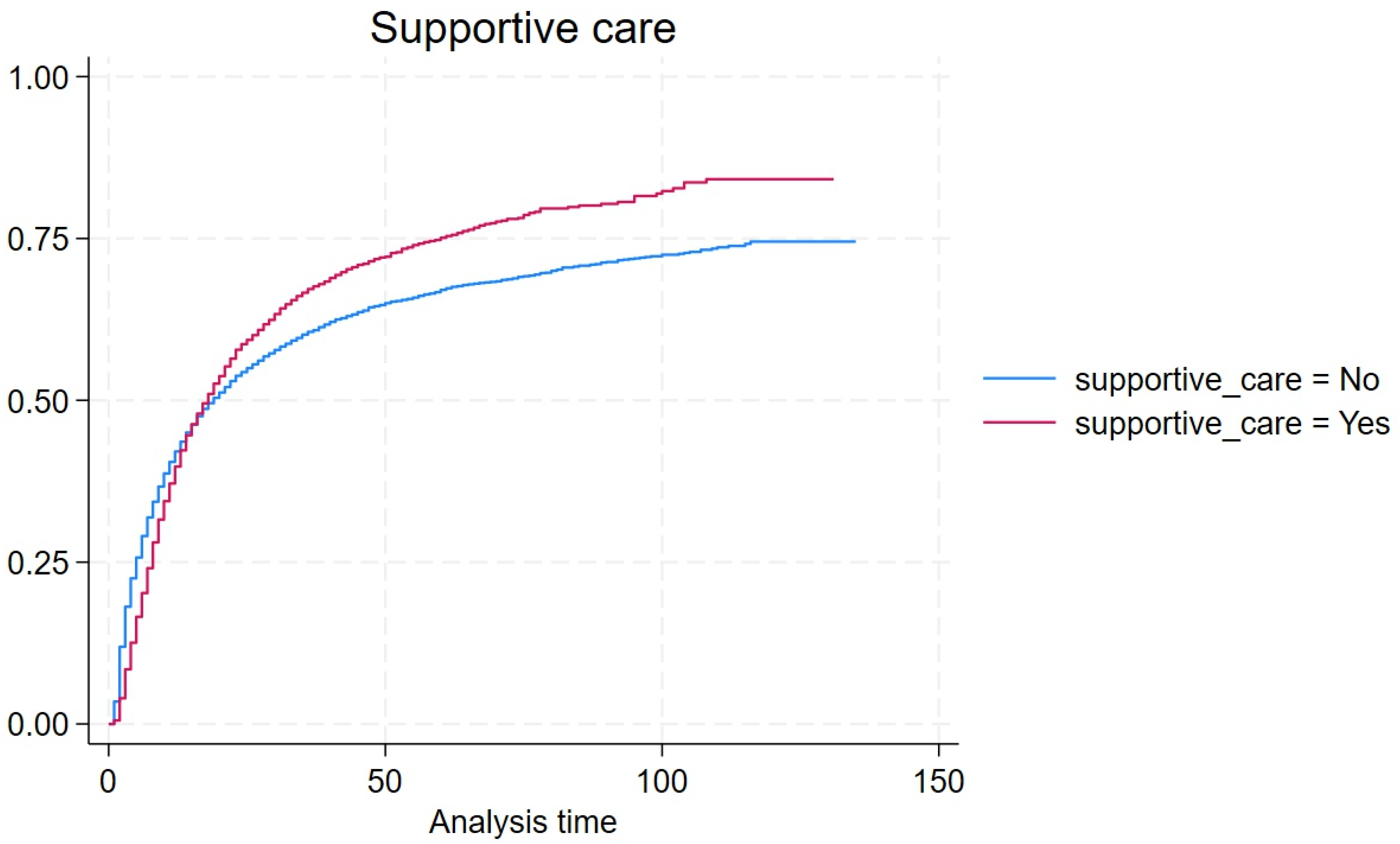

Supportive care
1.00
0.75
0.50
0.25
0.00
0
50
100
150
Analysis time
supportive_care = No
supportive_care = Yes

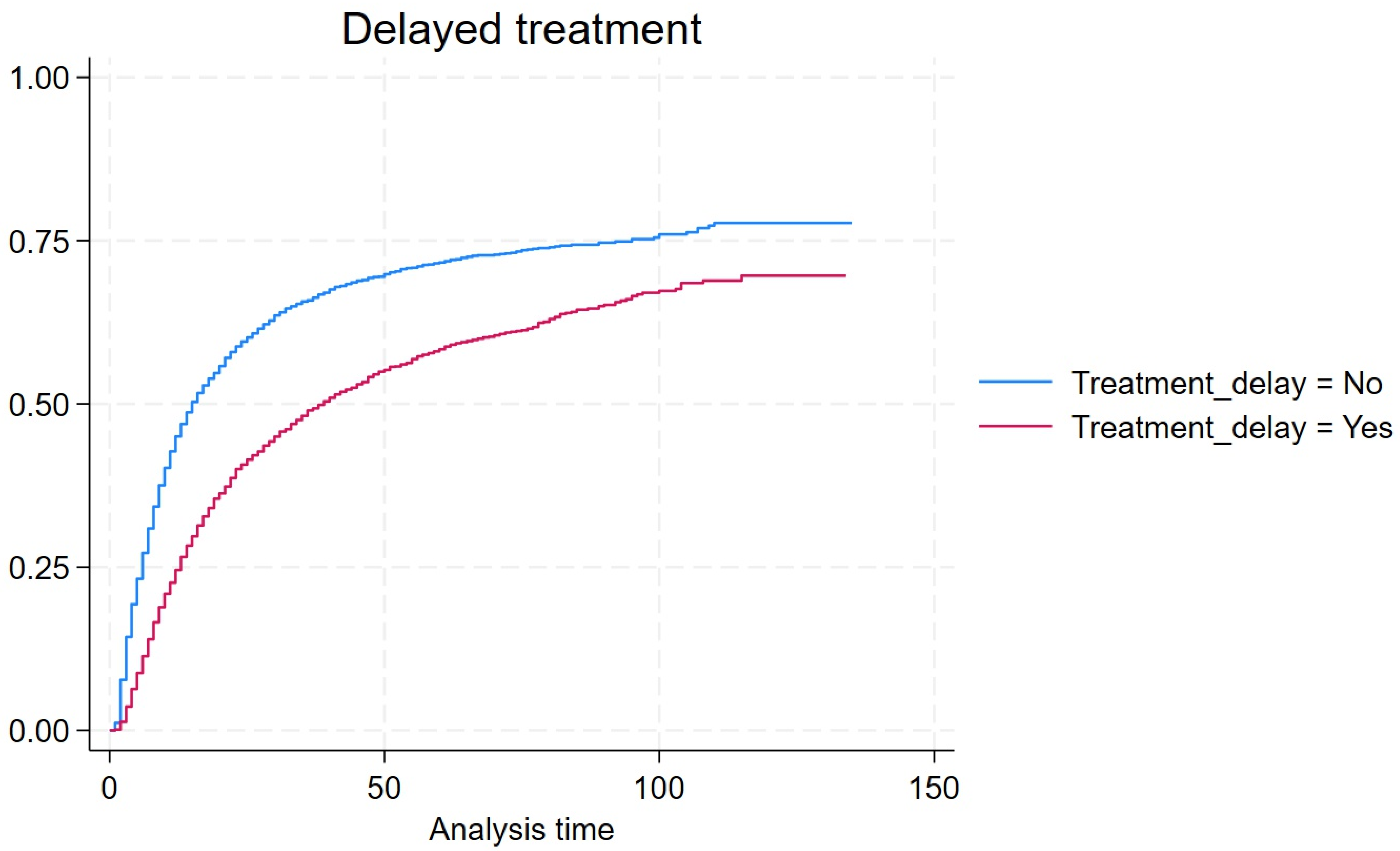
Delayed treatment
1.00
0.75
0.50
0.25
0.00
0
50
100
150
Analysis time
Treatment_delay = No
Treatment_delay = Yes

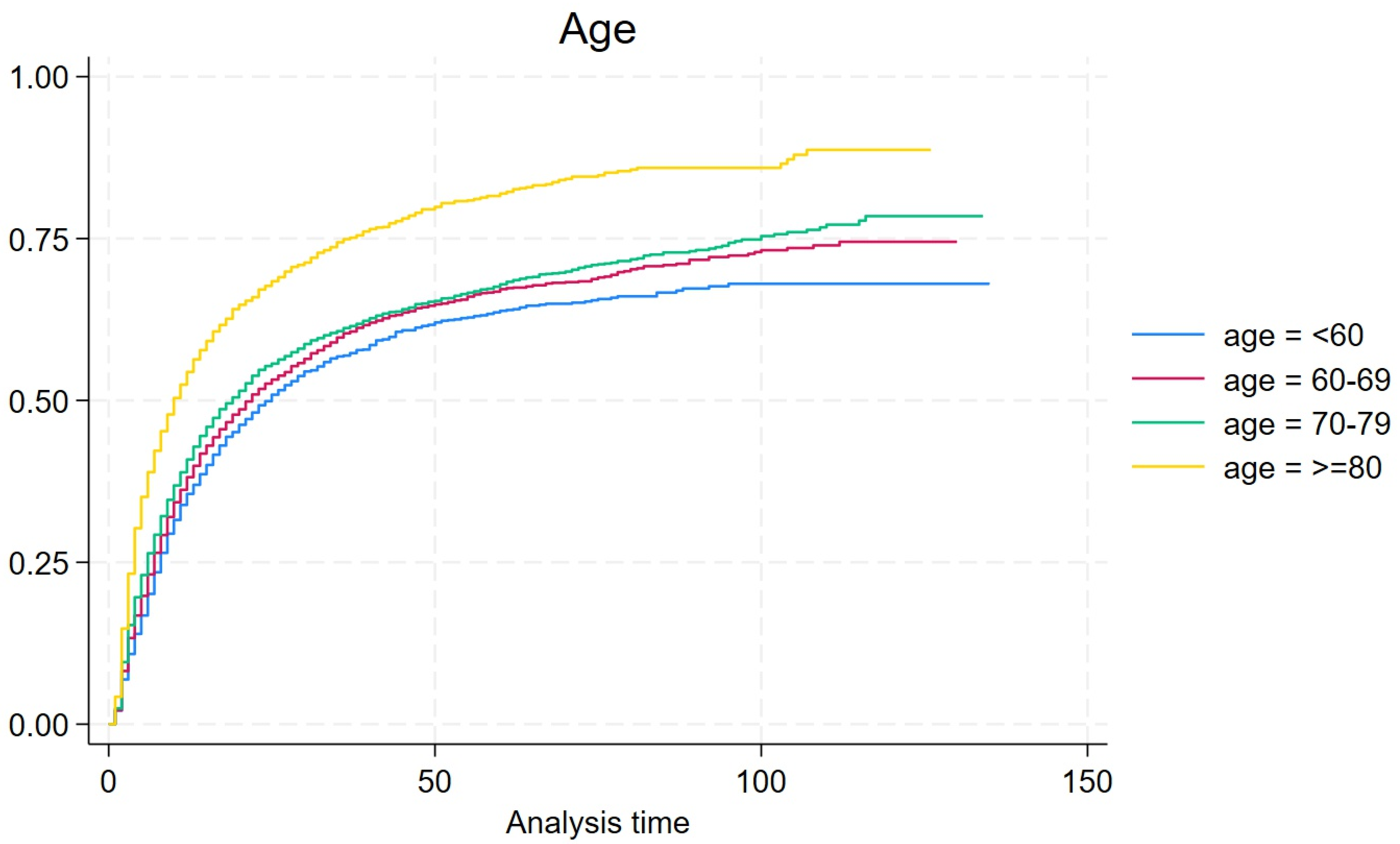
Age
1.00
0.75
0.50
0.25
0.00
0
50
100
150
Analysis time
age = <60
age = 60-69
age = 70-79
age = >=80

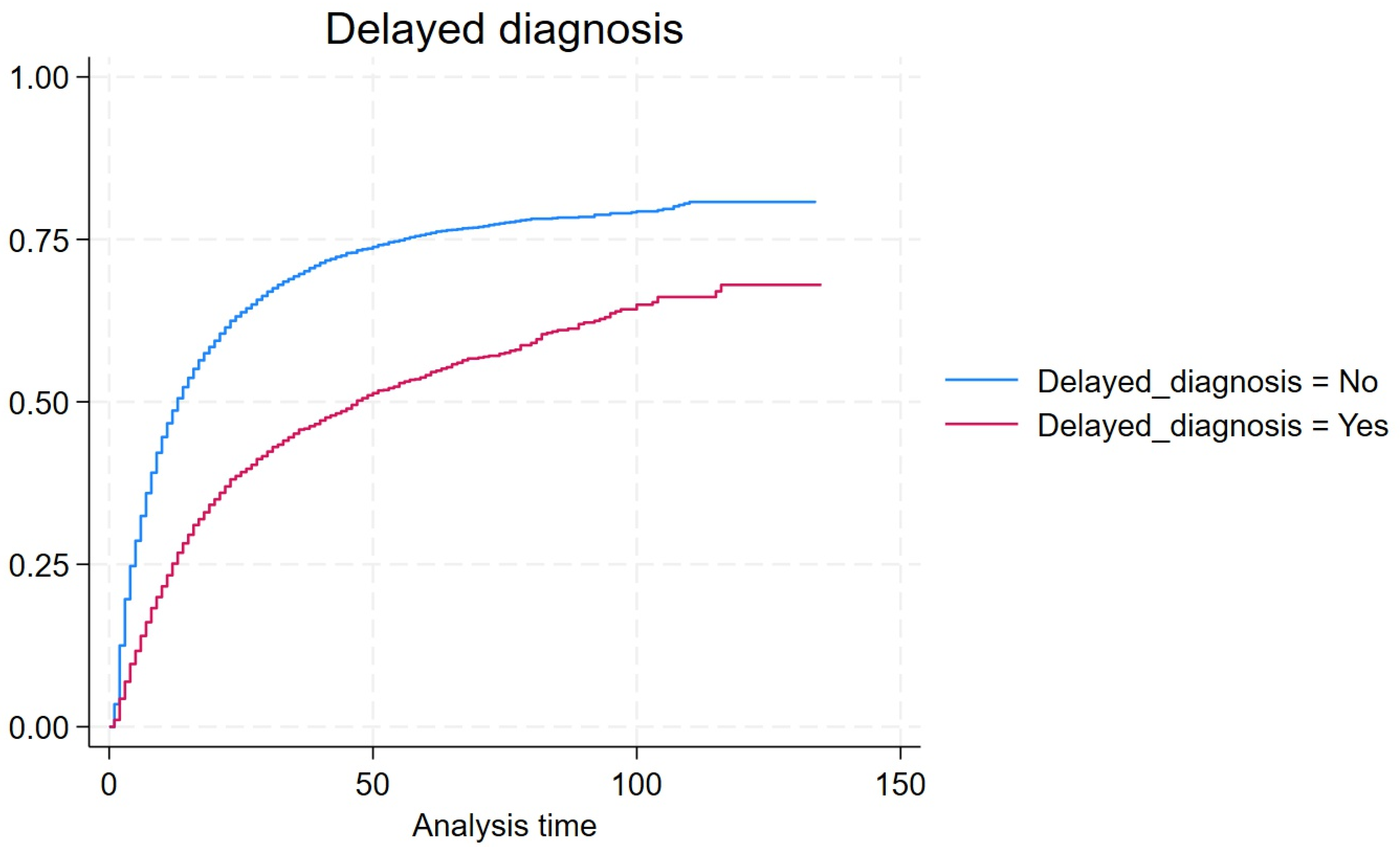

Delayed diagnosis
1.00
0.75
0.50
0.25
0.00
0
50
100
150
Analysis time
Delayed_diagnosis = No
Delayed_diagnosis = Yes

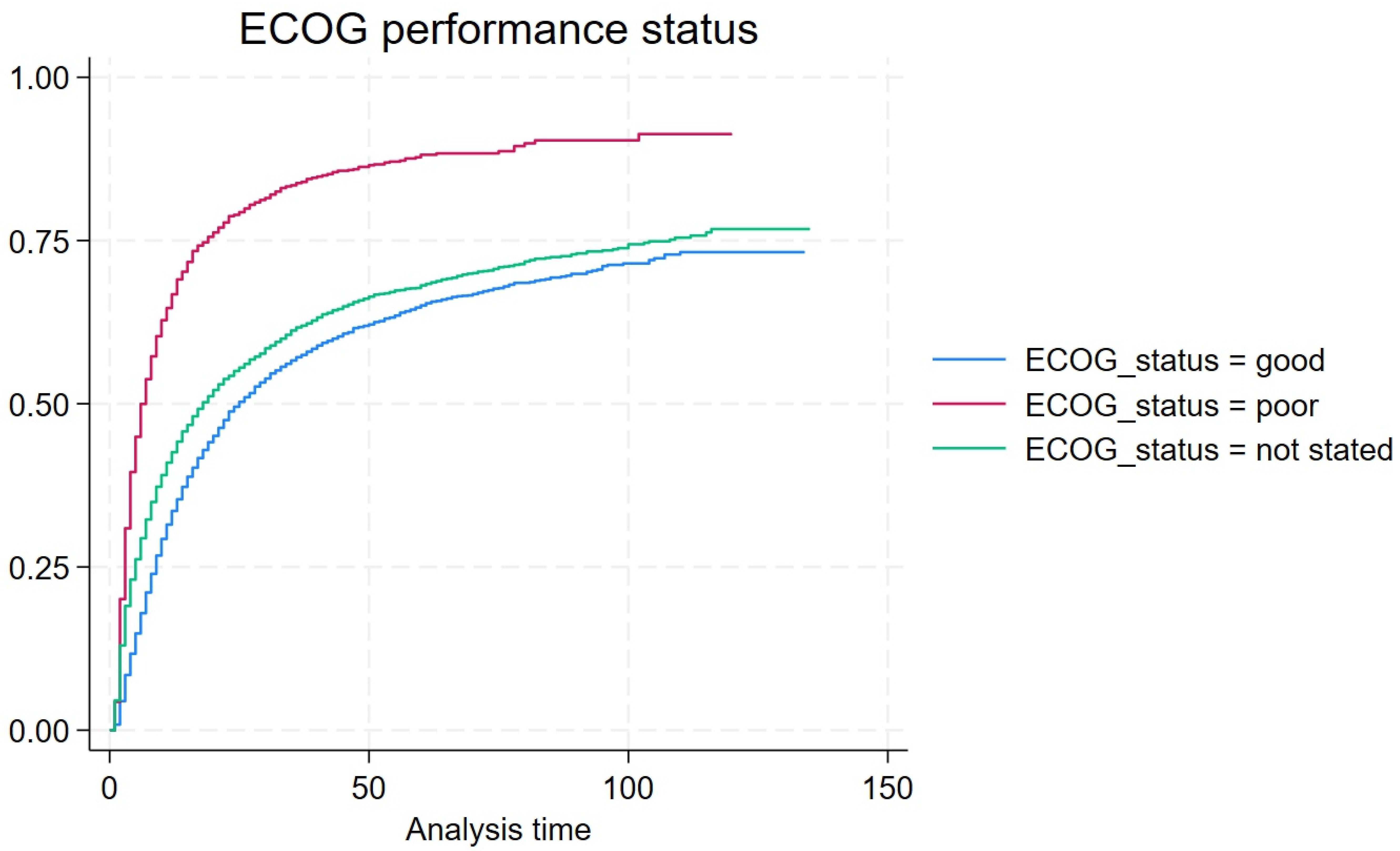
ECOG performance status
1.00
0.75
0.50
0.25
0.00
0
50
100
150
Analysis time
ECOG_status = good
ECOG_status = poor
ECOG_status = not stated

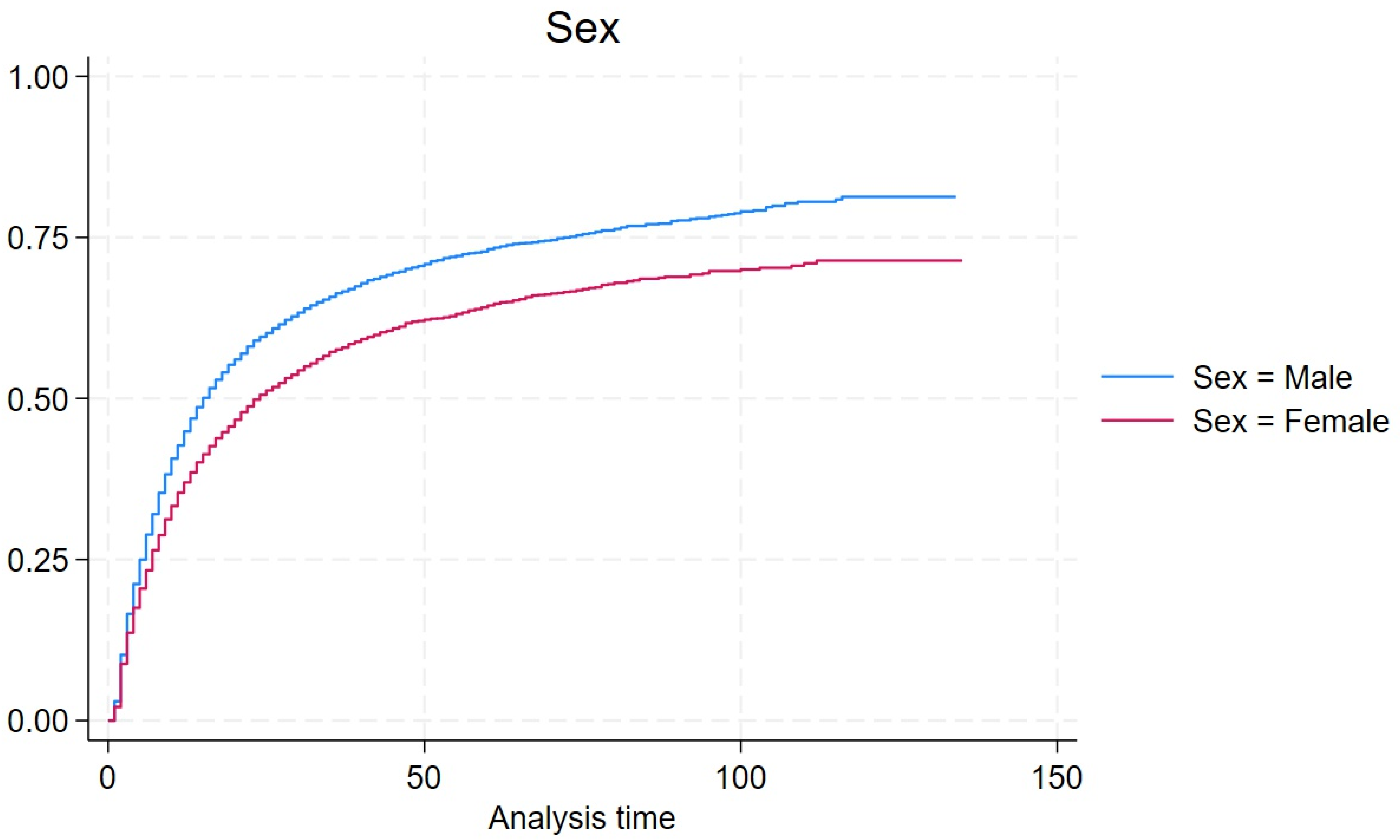

Sex
1.00
0.75
0.50
0.25
0.00
0
50
100
150
Analysis time
Sex = Male
Sex = Female

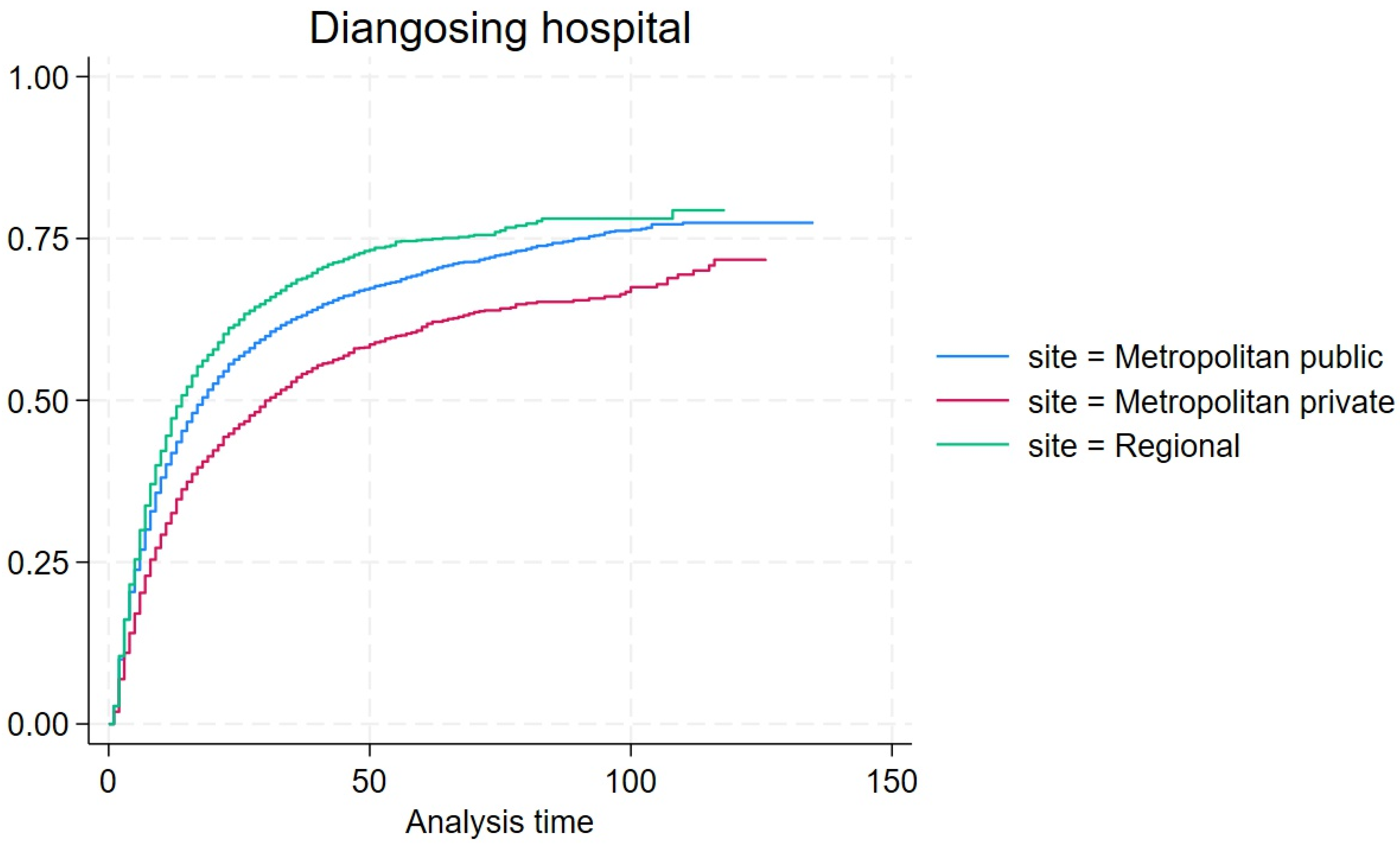

Diangosing hospital
1.00
0.75
0.50
0.25
0.00
0
50
100
150
Analysis time
site = Metropolitan public
site = Metropolitan private
site = Regional
