# Supplementary material for: Patient‐ and Areal‐Level Risk Factors Associated With Lung Cancer Mortality in Victoria, Australia: A Bayesian Spatial Survival Analysis
Source: Cancer Med. 2024 Oct 9;13(19):e70293. doi: 10.1002/cam4.70293 (PMC11462597; doi:10.1002/cam4.70293)
Supplement: Supplementary file 2 — Figure S2. [file CAM4-13-e70293-s003.pdf]

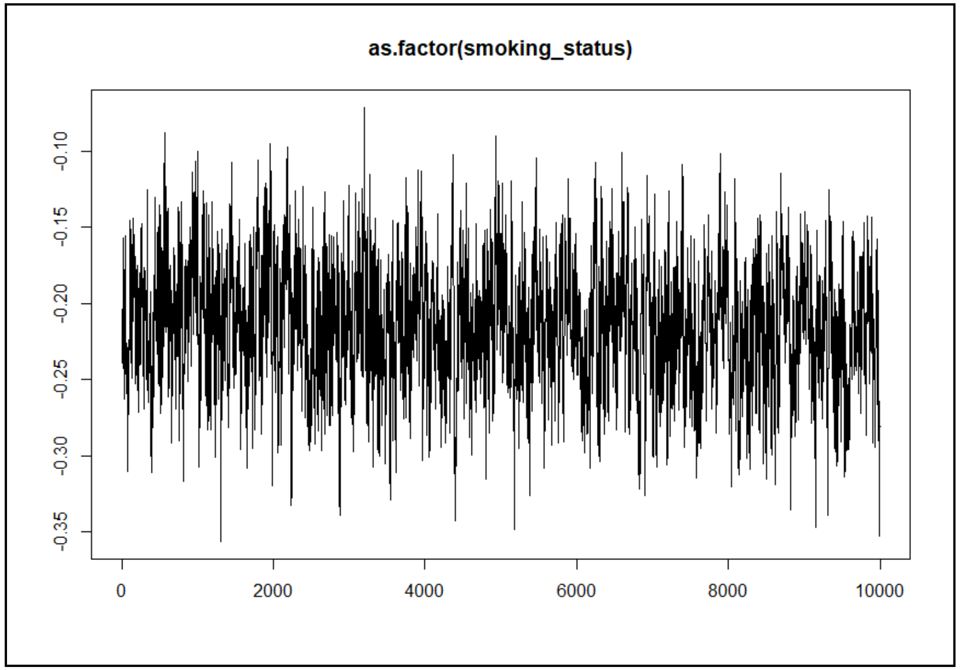
as.factor(smoking_status)
-0.10 -0.15 -0.20 -0.25 -0.30 -0.35
0 2000 4000 6000 8000 10000

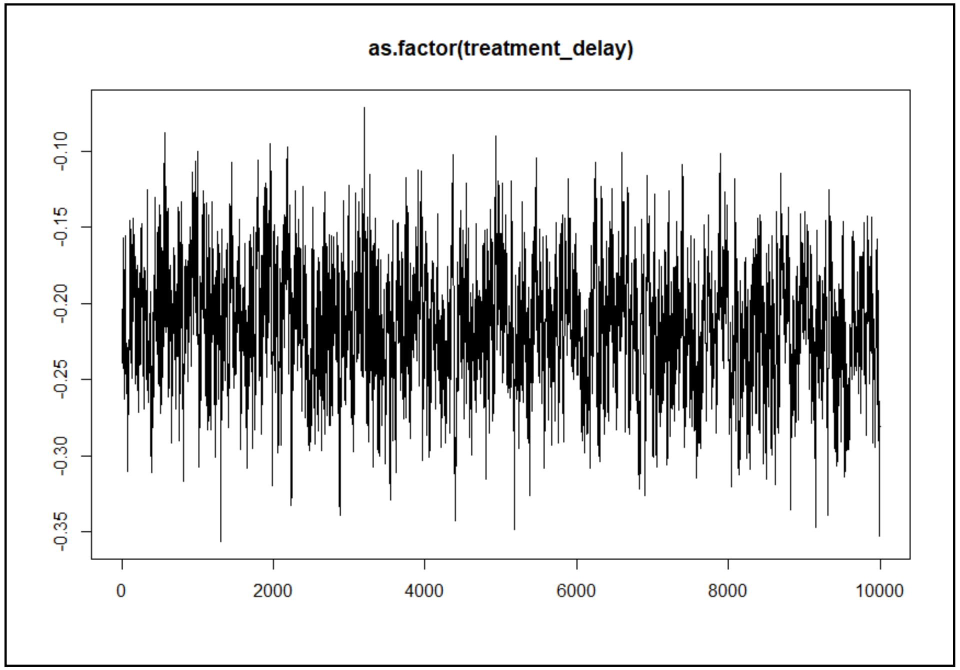
as.factor(treatment_delay)
-0.10 -0.15 -0.20 -0.25 -0.30 -0.35
0 2000 4000 6000 8000 10000

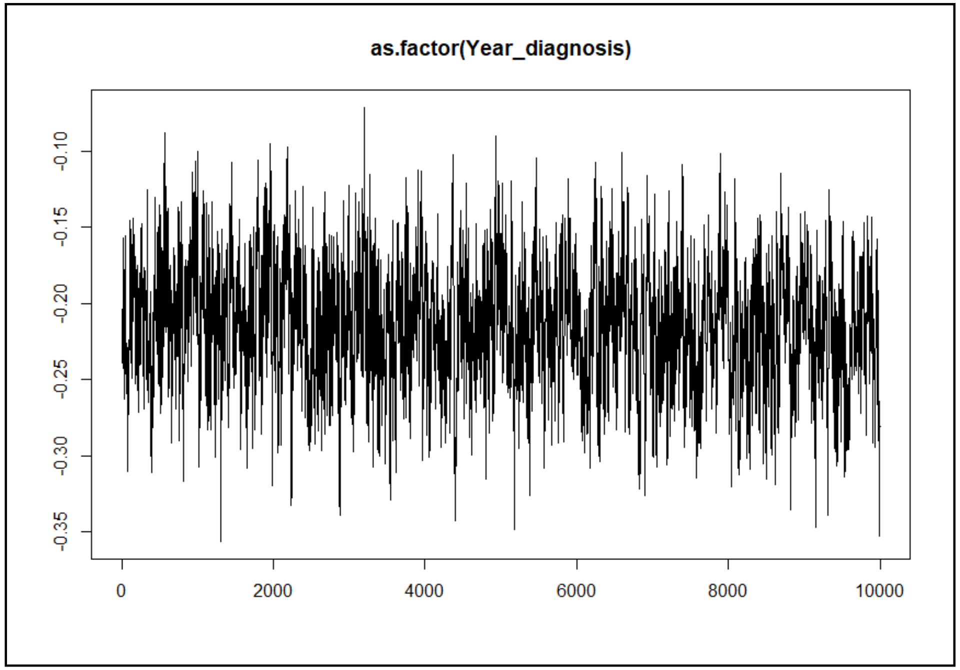
as.factor(Year_diagnosis)
-0.10 -0.15 -0.20 -0.25 -0.30 -0.35
0 2000 4000 6000 8000 10000

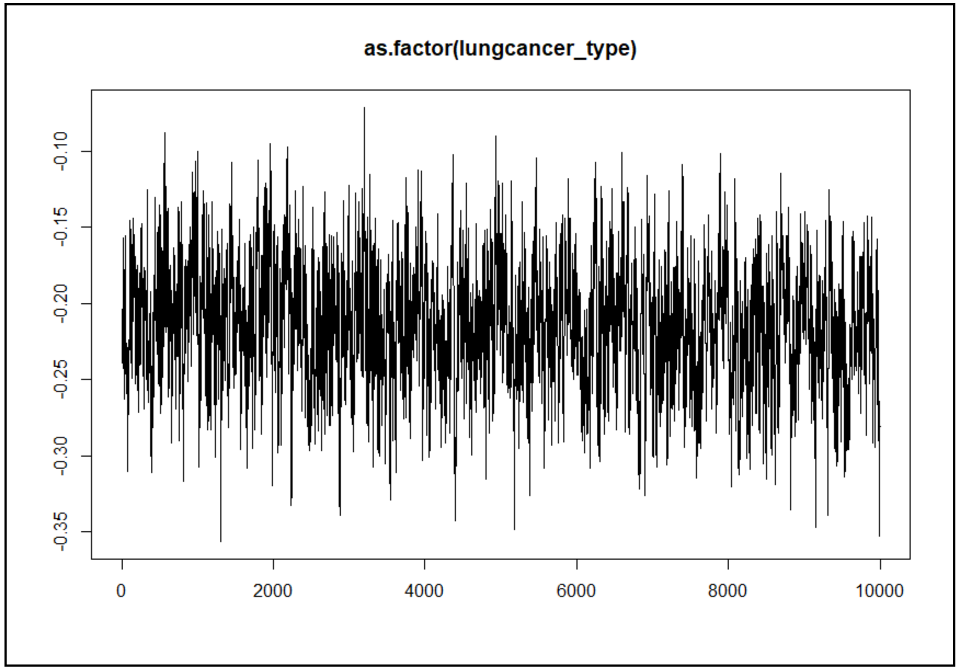
as.factor(lungcancer_type)
-0.10 -0.15 -0.20 -0.25 -0.30 -0.35
0 2000 4000 6000 8000 10000

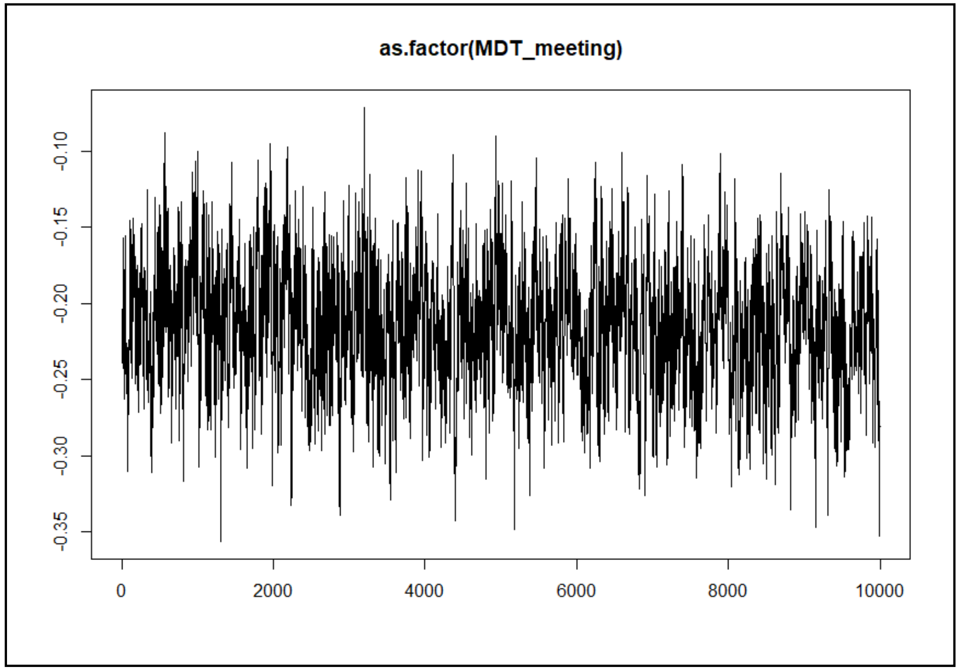
as.factor(MDT_meeting)
-0.10
-0.15
-0.20
-0.25
-0.30
-0.35
0
2000
4000
6000
8000
10000

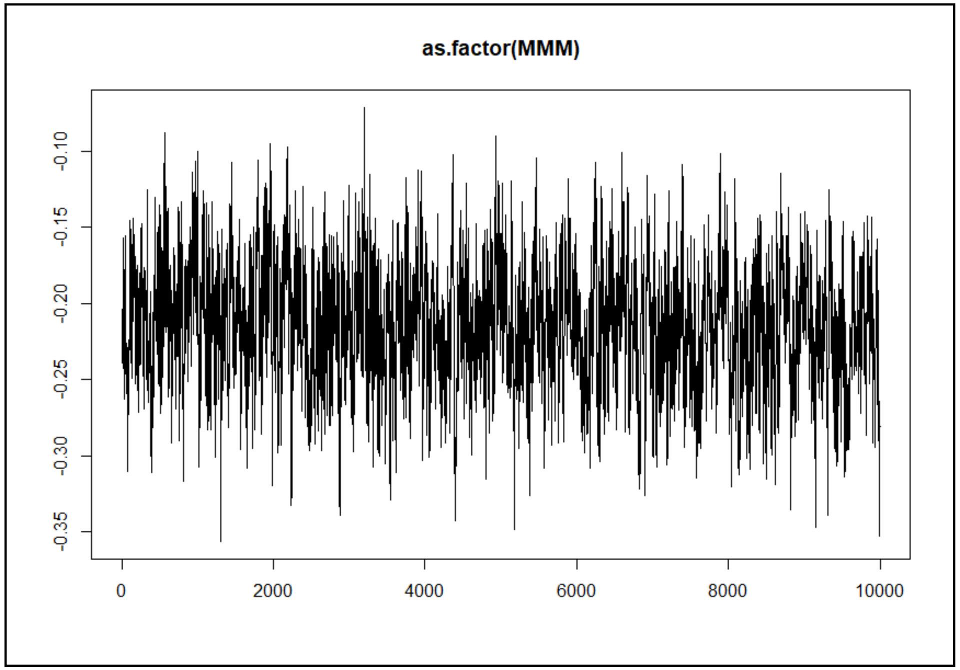
as.factor(MMM)
-0.10
-0.15
-0.20
-0.25
-0.30
-0.35
0
2000
4000
6000
8000
10000

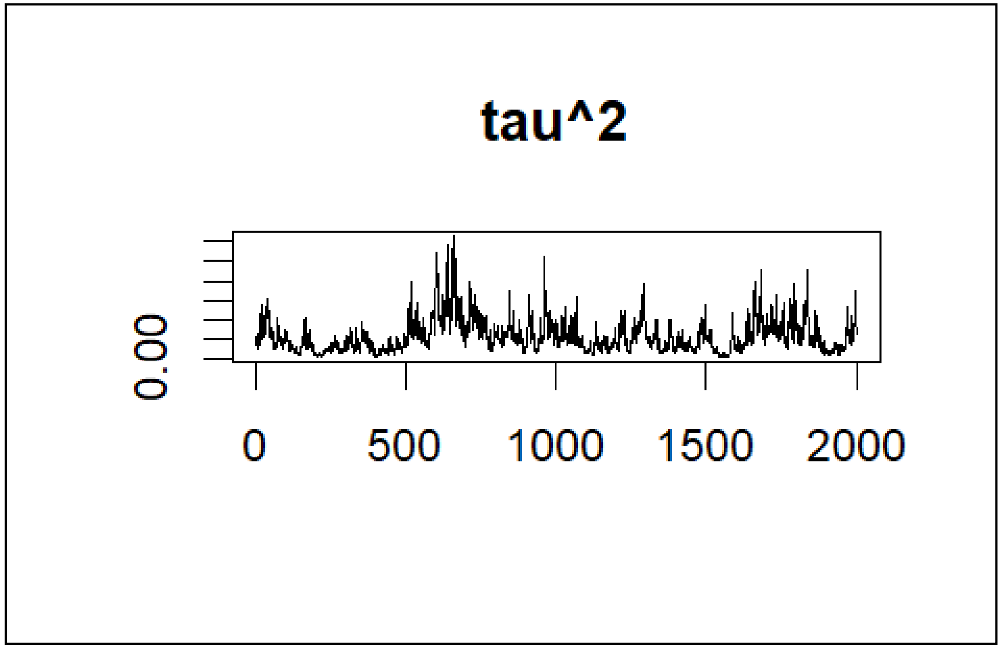
tau^2
0.00
0
500
1000
1500
2000

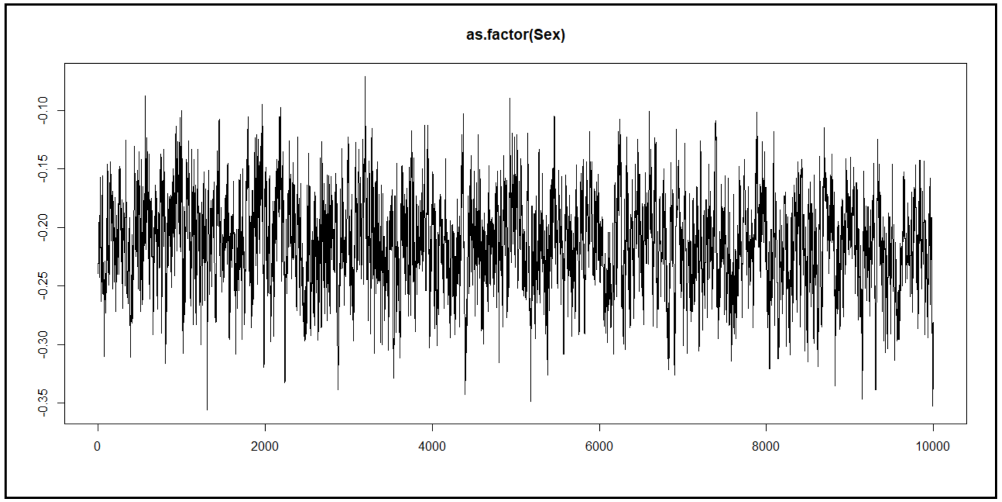
as.factor(Sex)
-0.10
-0.15
-0.20
-0.25
-0.30
-0.35
0
2000
4000
6000
8000
10000

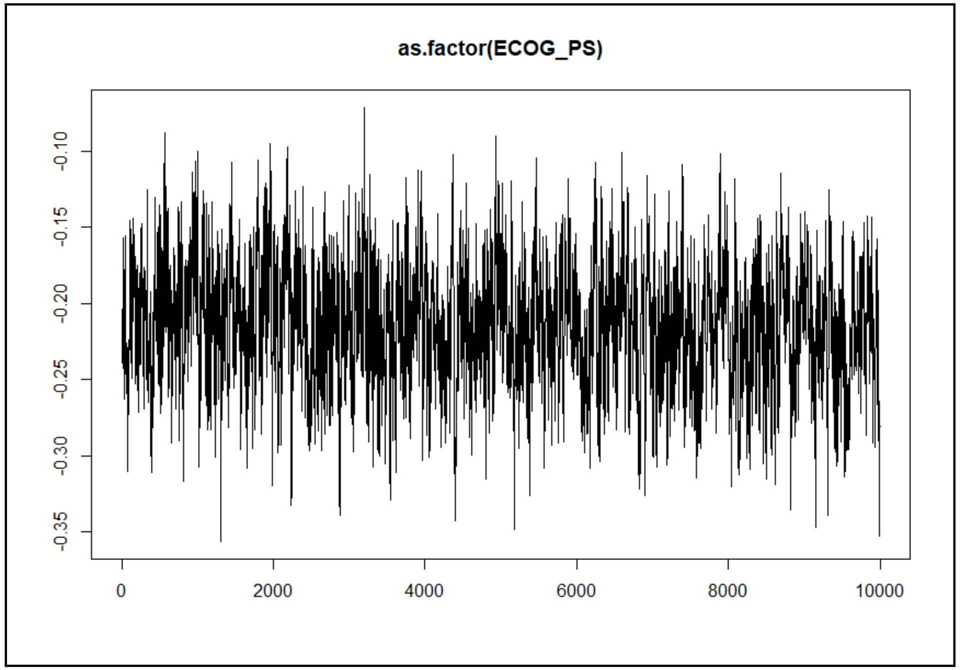
as.factor(ECOG_PS)
-0.10
-0.15
-0.20
-0.25
-0.30
-0.35
0
2000
4000
6000
8000
10000

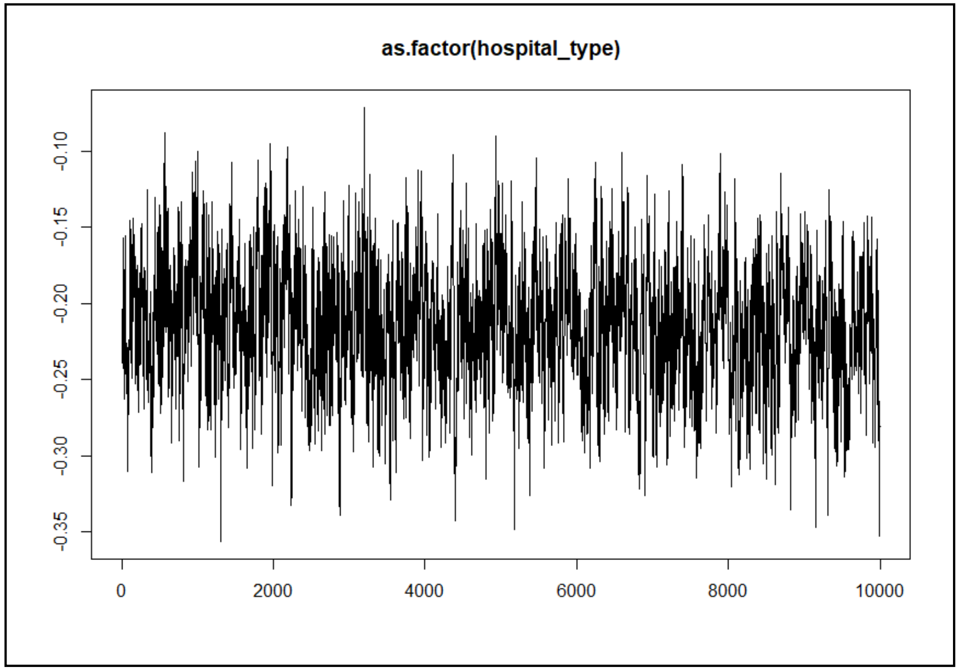
as.factor(hospital_type)
-0.10
-0.15
-0.20
-0.25
-0.30
-0.35
0
2000
4000
6000
8000
10000

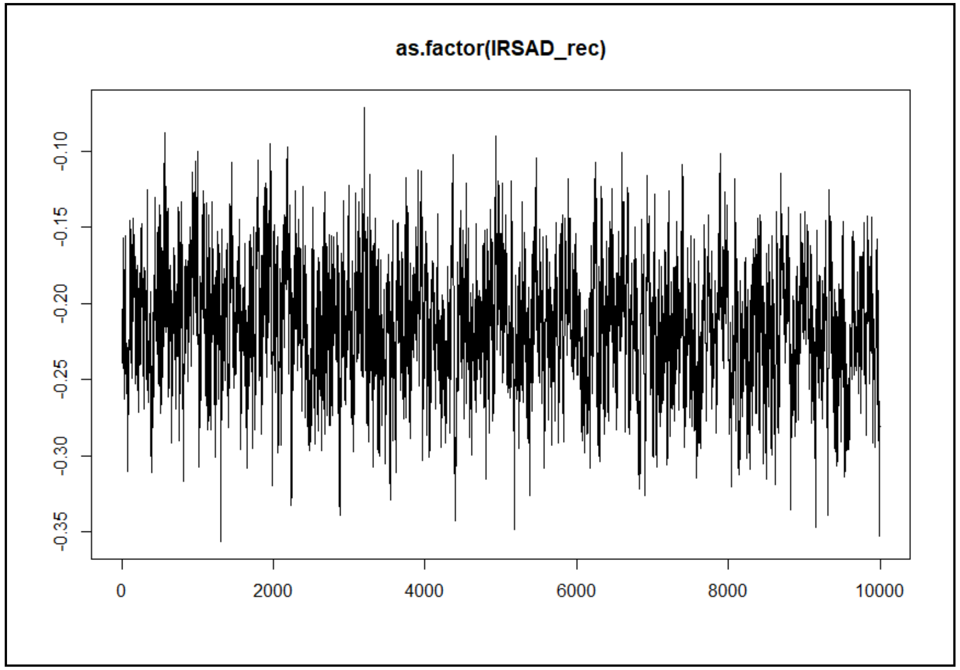
as.factor(IRSAD_rec)
-0.10
-0.15
-0.20
-0.25
-0.30
-0.35
0
2000
4000
6000
8000
10000

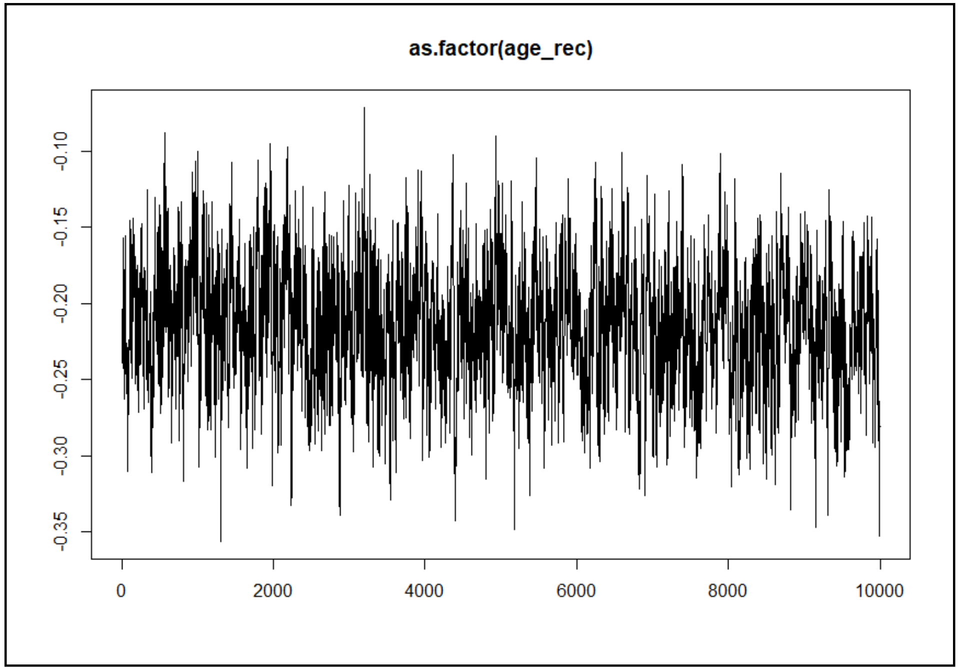
as.factor(age_rec)
-0.10
-0.15
-0.20
-0.25
-0.30
-0.35
0
2000
4000
6000
8000
10000

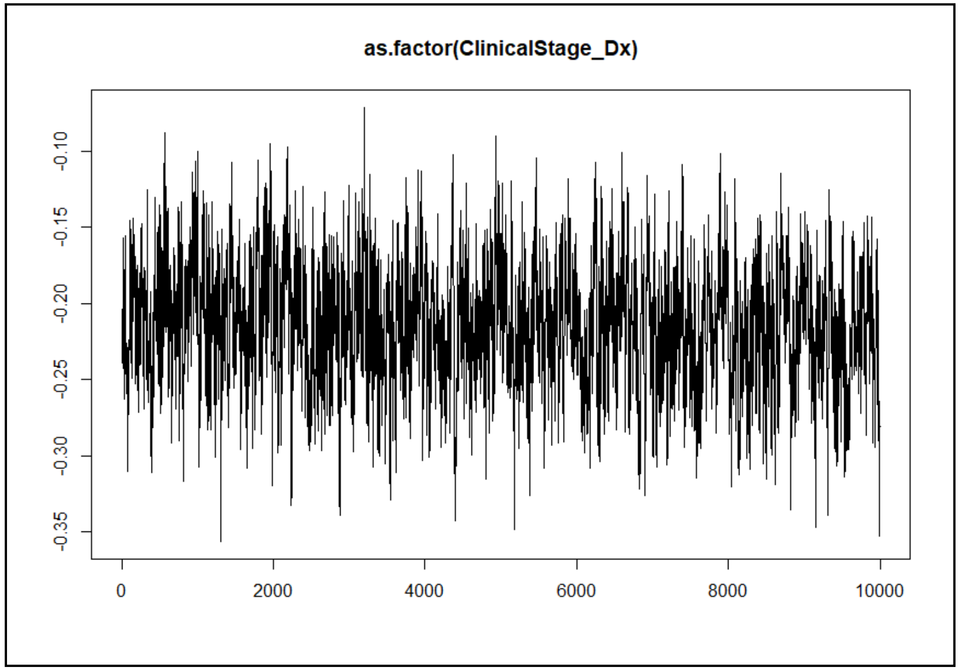
as.factor(ClinicalStage_Dx)
-0.10
-0.15
-0.20
-0.25
-0.30
-0.35
0
2000
4000
6000
8000
10000

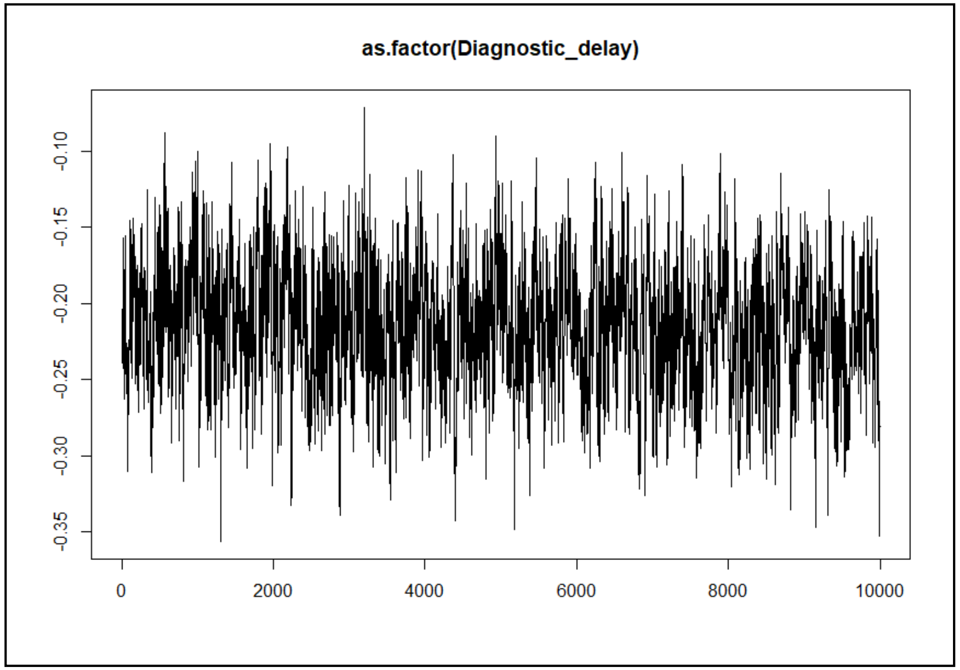
as.factor(Diagnostic_delay)
-0.10
-0.15
-0.20
-0.25
-0.30
-0.35
0
2000
4000
6000
8000
10000
