# Supplementary figures and images for: Patient‐ and Areal‐Level Risk Factors Associated With Lung Cancer Mortality in Victoria, Australia: A Bayesian Spatial Survival Analysis
Source: Cancer Med. 2024 Oct 9;13(19):e70293. doi: 10.1002/cam4.70293 (PMC11462597; doi:10.1002/cam4.70293)

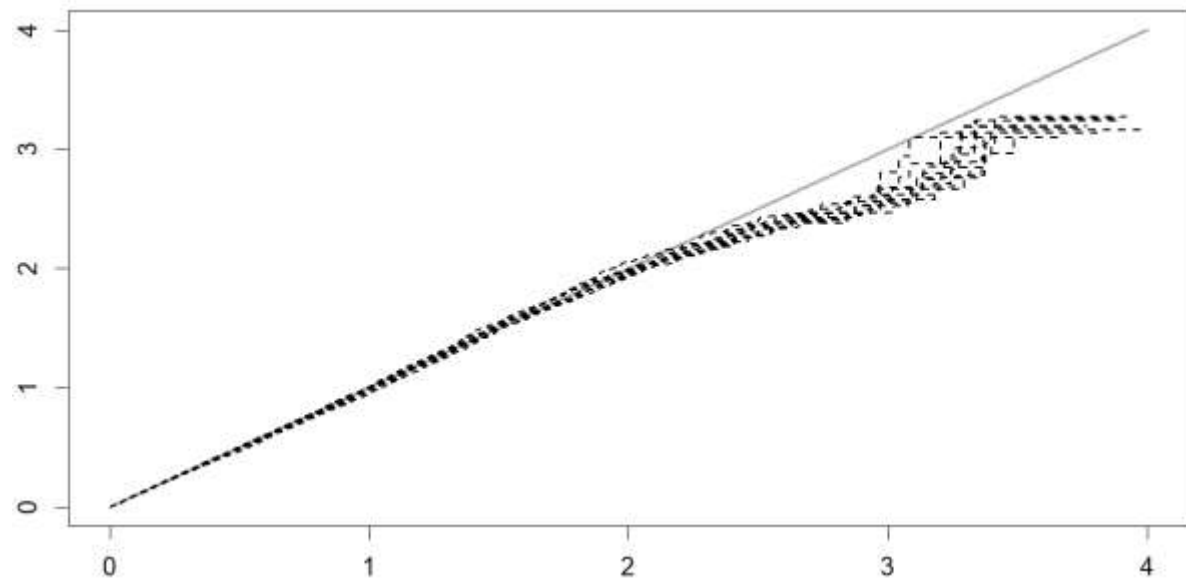

Figure S3: Cox–Snell residuals for the spatial log-logistic frailty model of Victoria

Supplement: Supplementary file 3 — Figure S3. [file CAM4-13-e70293-s001.pdf]
