# Supplementary material for: Patient‐ and Areal‐Level Risk Factors Associated With Lung Cancer Mortality in Victoria, Australia: A Bayesian Spatial Survival Analysis
Source: Cancer Med. 2024 Oct 9;13(19):e70293. doi: 10.1002/cam4.70293 (PMC11462597; doi:10.1002/cam4.70293)
Supplement: Supplementary file 4 — Data S1. [file CAM4-13-e70293-s002.docx]

**Table S1: Comparison of survival distribution and frailty term**

| **Model** | LPML | DIC | WAIC |
| --- | --- | --- | --- |
| Null model without frailty | -36576.27 | 73152.54 | 73152.54 |
| Null model with CAR frailty | -36567.76 | 73135.48 | 73135.52 |
| Spatial log-normal model | -33688.85 | 67376.23 | 67377.72 |
| Spatial Weibull model | -34098.6 | 68189.28 | 68197.17 |
| Spatial log-logistic model | -33727.67 | 67454.54 | 67455.35 |

^**^*Abbreviation: CAR = Conditional Autoregressive, DIC = Deviance Information Criterion, LPML = Log Pseudo Marginal Likelihood, WAIC = Watanable Akaike Information Criterion*

**Table S2: Posterior CAR variance of the final best fitted model**

| CAR frailty | Mean | Median | Std Deviation | 95% lower CI | 95% upper CI |
| --- | --- | --- | --- | --- | --- |
| Variance | 0.01507 | 0.1219 | 0.01142 | 0.00182 | 0.04500 |

^**^*Abbreviation: CAR = Conditional Autoregressive*
